# Low-affinity exchangeable probes for super-resolution and long-term fluorescence imaging

**DOI:** 10.1039/d6sc05417j

**Published:** 2026-10-22

**Authors:** Andrey S. Klymchenko, Remi Pelletier, Pascal Didier

**Affiliations:** a Laboratoire de Bioimagerie et Pathologies, UMR 7021 CNRS, ITI SysChem-Chimie des Systèmes Complexes, Université de Strasbourg 74 route du Rhin 67401 Illkirch France andrey.klymchenko@unistra.fr

## Abstract

To overcome the limitations of conventional fluorescence labelling based on covalent bonds or strong probe–target interactions, low-affinity labelling has recently emerged as an attractive alternative. Owing to the rapid exchange of probes at the labelling site, it circumvents two major challenges: it enables virtually unlimited labelling with minimal photobleaching and provides a simple mechanism for probe ON/OFF switching, which is essential for super-resolution imaging. This review covers the main classes of low-affinity probes for biological targets. The first major class is based on lock-and-key interactions between fluorophores and their targets, such as biomembranes, RNA aptamers, protein tags and endogenous biomolecules. In these systems, the formation of dye–target complexes generally induces a fluorogenic response, enabling imaging with low background despite the presence of a large pool of unbound probes. The second class relies on fluorescently labelled ligands that specifically recognize biological targets. The primary example is DNA-PAINT, which exploits the reversible hybridization of a short fluorescently labelled oligonucleotide with its complementary counterpart attached to a target-specific vector (*e.g.*, antibodies, nanobodies, or affimers). Emerging approaches also employ peptides, proteins, nucleic acid aptamers, and small molecules as low-affinity ligands. Low-affinity labelling already enables imaging with resolution down to ∼1 nm, but it remains rather slow and mainly compatible with fixed cells. We anticipate that this field will continue to expand rapidly, driven on the one hand by new concepts in fluorogenic dye design and reversible labelling strategies and, on the other hand, by the growing range of applications in advanced fluorescence microscopy.

## Abstract 2

Low-affinity fluorescence labelling can overcome the limitations of conventional labelling based on covalent bonds. Owing to the rapid probe exchange, it enables virtually unlimited recording time and ON/OFF switching for super-resolution imaging.

## Graphical abstract

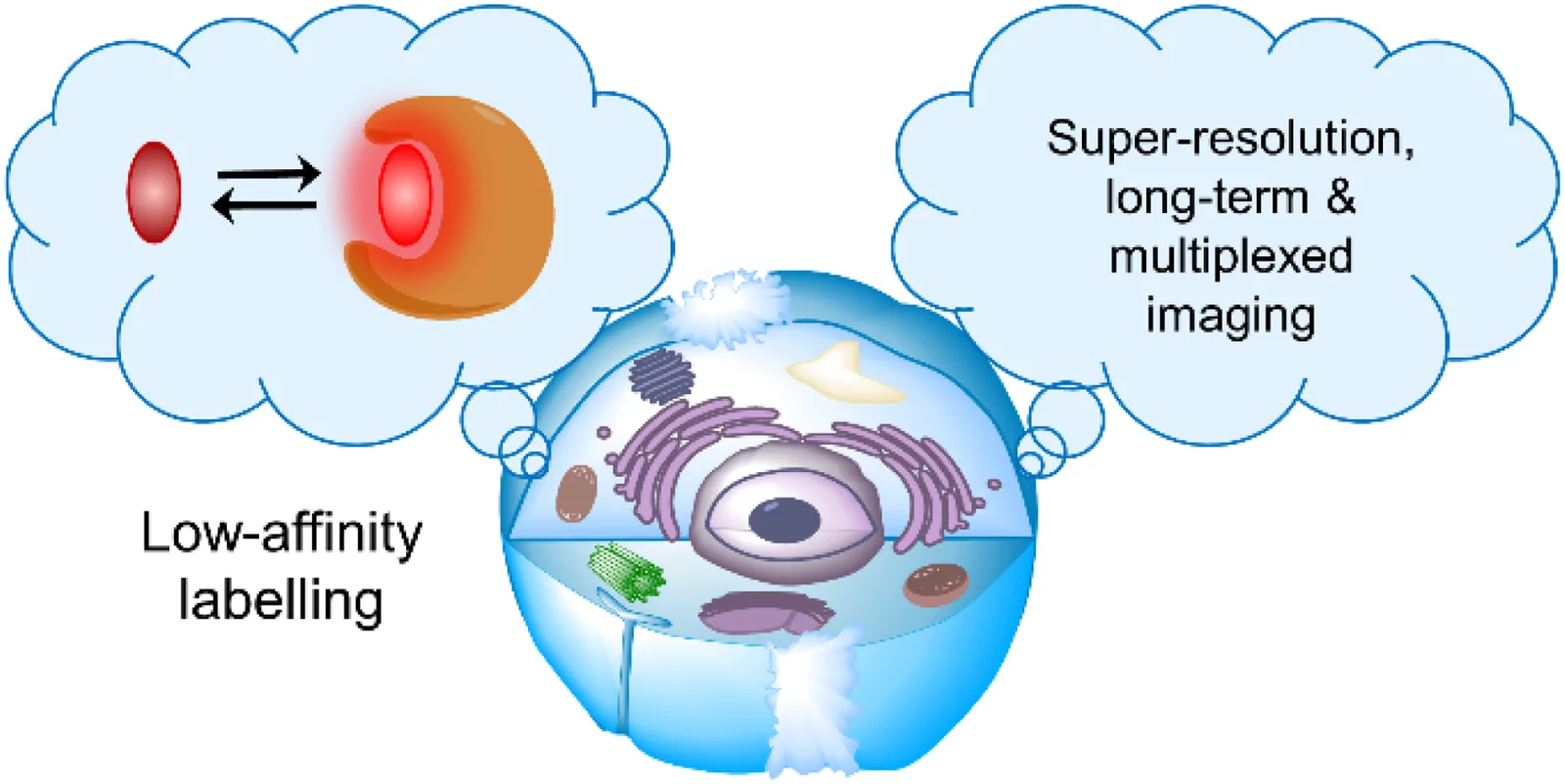

## 1. Introduction

Currently, fluorescence labelling and imaging rely mainly on covalent or high-affinity labelling. These are traditional approaches which are robust due to the firm link between the label and a biomolecule. However, they reach their limits when it comes to advanced fluorescence microscopy. First of all, fluorophores photobleach and produce reactive oxygen species.^1–3^ Therefore, biological molecules can be monitored for rather limited time, while the long-term observations lead to signal loss and phototoxicity. A number of methods were recently proposed to improve the photostability of fluorophores, such as the use of reducing and oxidative reagents (ROX)^4^ and triplet quenchers,^5,6^ the use of plasmonic effects,^7,8^ near-infrared light to deactivate the triplet state,^9^ tailor made fluorophore design,^10–14^ protection of the dyes within macrocycles,^15–17^*etc.* Many of these methods had also significantly decreased phototoxicity, allowing longer recording with decreased photodamage. Nevertheless, they remain physically limited in terms of the number of photons a single molecule can deliver before photobleaching, which rarely exceeds 10^6^ photons.

On the other hand, the last decade has seen a remarkable rise from single-molecule localization microscopy (SMLM), which enabled imaging biological systems with superior resolution down to nanometres^18–20^ and even to ångströms.^21,22^ Among them, methods based on single-molecule localization microscopy systematically require dyes capable of undergoing ON/OFF switching, which is challenging with classical dyes because they usually do not show this behaviour. Therefore, dedicated approaches have been established to implement (photo)switching behaviour, which includes tailor made design of new fluorophores,^23–25^ the use of special blinking media^26,27^ and combination of dye pairs.^28^

A completely different concept that can address the fundamental problems of dye photostability, phototoxicity and switching is to replace covalent labelling with a reversible low-affinity labelling. In this case, the probe is no more attached to the target, but binds it reversibly during the course of the measurement. Low affinity to the target is associated with a high unbinding rate (*k*_OFF_), which ensures that the same molecule stays for limited time at the binding site. Moreover, the binding rate (*k*_ON_) should also be sufficiently high (ideally close to the diffusion rate) to ensure rapid replacement with a new molecule. As the probe can be continuously renewed at the binding site, in principle, it provides “eternal” labelling. Even though this approach can resolve the problem of photostability, it does not address directly the phototoxicity issues because the dye renewal does not stop generation of singlet oxygen, which is often regarded as the major cause of the phototoxic effect of dyes. Nevertheless, low affinity also means low local concentration of the dye and thus a lower rate of singlet oxygen generation, which should potentially decrease phototoxicity, as shown previously.^29^ On the other hand, reversible binding can be directly associated with ON/OFF switching of the dyes on target binding and their localization, which can be used in super-resolution imaging. In 2006, Hochstrasser and co-workers introduced a technique called PAINT,^30^ which used fluorogenic and solvatochromic dye Nile red that is poorly emissive in water but highly emissive after binding to the lipid membrane. The dynamic binding–unbinding of Nile red provided dye immobilization and an ON/OFF switching mechanism that allowed super-resolution PAINT imaging of biological membranes. The PAINT principle was further extended to reversible hybridization of a short fluorescently labelled DNA oligonucleotide (DNA-PAINT),^31,32^ proteins^33–35^ and other ligands.^36^ In DNA-PAINT the dye is “immobilized” on the target through a DNA ligand, which provides an apparent turn-ON of the signal. The latter has a limitation of non-negligible background from always ON unbound probe species. Therefore, recent research has focused on development of new turn-ON mechanisms to ensure faster PAINT with higher signal-to-noise ratios.^37,38^ Finally, an important aspect is an increase in the information content when using exchangeable probes, which includes spectral resolution^39,40^ molecular diffusion^41^ and orientation.^42^

Overall, the development of exchangeable probes for labelling biological systems and corresponding imaging methods constitute a rapidly expanding research direction in chemical biology. The present review will cover major aspects of chemical design of exchangeable probes and the methods of their imaging in order to obtain information about living systems with superior resolution, minimal phototoxicity and maximal information content.

## 2. Low-affinity labelling: principles

Low-affinity exchangeable probes are fluorescent molecules composed of a fluorescent dye and a targeting group, characterized by high specificity but weak reversible binding to the target. This approach is different from covalent methods where specificity is achieved by a chemical reaction resulting in a stable conjugate that cannot reversibly exchange. The key challenge in the design of reversible labels is the combination of high specificity of labelling with its reversibility. This could be achieved by exploiting the molecular recognition principle by a lock-and-key principle, where the ligand (key) could reversibly and selectively bind to the (biological) target (lock). The existing exchangeable probes could be split into those based on fluorophore or ligand recognition (Fig. 1). The key difference between these classes is that in the former the fluorophore itself plays the role of the recognition ligand, while the latter uses a dedicated recognition ligand that is conjugated to a fluorophore, not participating in the recognition processes.

**Fig. 1:**
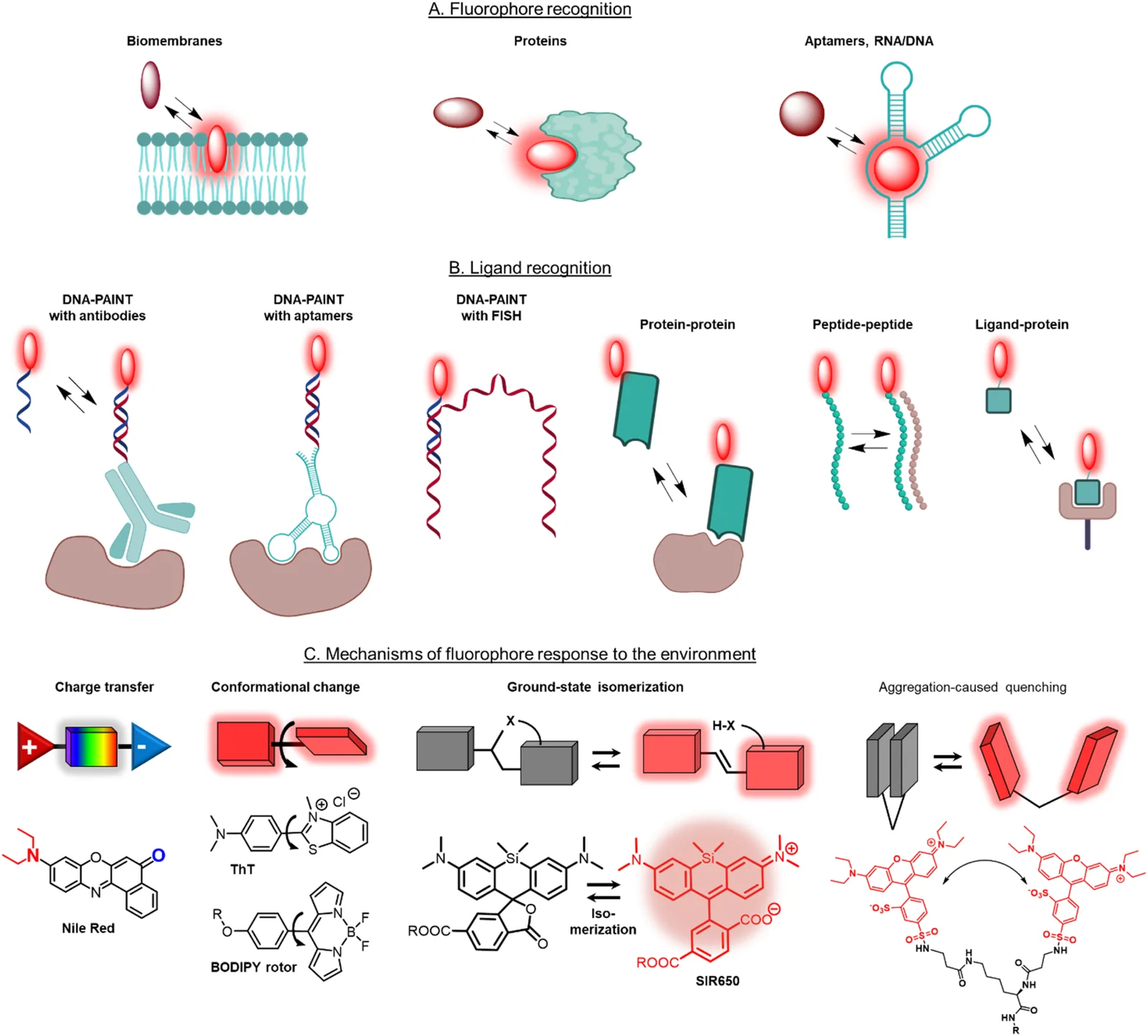
Reversible labelling of biological targets. (A) Probes based on fluorophore recognition using biological membranes, RNA aptamers and engineered proteins. (B) Probes based on ligand recognition, where reversible labelling is provided by the DNA hybridization principle (DNA-PAINT), while recognition ligands are antibodies, aptamers and DNA (using the FISH method). Peptide/protein-based ligands using peptide coiled–coil recognition and antibody fragment-antigen recognition; small-molecule ligand–receptor recognition. (C) The key mechanisms involved in the fluorogenic and chromogenic response of dyes to the local environment.

Let us present briefly some types of exchangeable probes based on fluorophore recognition. The primary type exploits interactions between the fluorophore and lipid membranes (Fig. 1A), representing the first concept proposed for PAINT super-resolution imaging in 2006.^30^ In this case, Nile red has an intrinsic lipophilic property (high log *P*), which ensures its affinity to lipid membranes. However, in cells Nile red lacks targeting specificity, because it can bind to a multitude of biological membranes, including plasma membranes and intracellular membranes of organelles, as well as to lipid droplets. Therefore, dedicated design strategies were proposed to improve specificity of membrane labelling using low affinity membrane anchors.^43^

The second type of probe is based on interaction of specially designed fluorophores with proteins (Fig. 1B). In this case the match between the dye molecule and a binding pocket of a given protein is achieved through both dye design and protein engineering. These probes are mainly represented by engineered protein tags, which could label any desired protein of interest. One example is the fluorescence-activating and absorption-shifting (FAST) protein tag, where the fluorogenic dye structurally similar to the GFP fluorophore lights up on binding to an engineered fluorescent protein FAST.^44^ Another key example is based on the HaloTag scaffold, which was originally proposed for covalent labelling with a fluorophore bearing chloroalkane ligand.^45^ In this case, the reactive HaloTag ligand was replaced with a non-covalent binder (xHTLs) which allowed exchange of the fluorescent ligand, while preserving high specificity.^33^

The third type exploits specific and reversible interaction of the fluorophore with RNA aptamers (Fig. 1C). The concept was originally proposed by Jaffrey and co-workers for fluorescent labelling of target RNA inside the cells.^46^ Those aptamers were specially designed to recognize a GFP-like fluorophore triggering its light up. Recently, low-affinity and high specificity were achieved for RNA aptamers, allowing PAINT-type super-resolution imaging of RNA in cells.^47^

The other big class of exchangeable probes is based on a dedicated recognition ligand conjugated to a fluorescent dye. It has gained enormous interest in the last decade since DNA hybridization was proposed by Jungmann *et al.* as a principle for low-affinity labelling, known as DNA-PAINT.^31,32^ In this case, labelling specificity is achieved due to base pairing of complementary sequences. The low affinity is provided by decreasing the length of the complementary strands to ∼8 bp, which is optimal to achieve a high unbinding rate (*k*_OFF_) at room temperature. This approach has been mainly used in combination with antibodies using DNA-antibody conjugates (Fig. 1D), which allows targeting practically any desired antigen within the cell. Further studies on DNA-PAINT have expanded the range of targetable biomolecules beyond antibodies. Examples include (a) nanobodies, which are single-domain antibody fragments with a smaller footprint,^48^ (b) aptamer-based probes with engineered recognition and DNA-hybridization sequences as well as slow-off-rate modified aptamers (SOMAmers), which are attractive alternatives because they can be directly used for reversible labelling with fluorescently labelled oligonucleotides (Fig. 1E),^49^ (c) fluorescence *in situ* hybridization (FISH) approaches that target specific cellular RNA transcripts by reversible hybridization with DNA oligonucleotides (Fig. 1F)^50^ and (d) DNA–lipid conjugates, which allow targeting and imaging of cell membranes.^51,52^

The emerging strategy of ligand-based low-affinity labelling relies on the use of peptides and protein fragments (Fig. 1G). One example is the use of the Fab domain of monoclonal antibody, displaying, in the presence of a chaotropic agent, low affinity to the target combined with good specificity.^53^ Another example of protein ligands includes specially screened intracellular binders of multiple intracellular targets.^54^ In addition, peptide ligands constitute an important emerging direction in low-affinity labelling that exploits transient coiled coil interactions with corresponding peptides/protein fragments within cell structures^55–57^ or short peptides that were shown to specifically target supramolecular hydrogel materials.^58^ Finally, one should also mention small molecule scaffolds as specific ligands for reversible labelling (Fig. 1H). In principle, they can cover practically any ligand–receptor interactions, which include synthetic drugs and natural molecules, like folic acid, phalloidin,^59^ carbohydrates,^60^*etc.* In the present review, all these low-affinity strategies, their specific chemistry and biological applications as well as methods for their detection will be explained.

Low-affinity labelling generally relies on two different types of fluorophores. Ligand-based strategies, including DNA hybridization, usually exploit classical fluorophores, which are always ON. This approach allows single-molecule localization microscopy by PAINT because immobilized fluorophores provide stronger signals than those freely diffusing in solution. However, it leads to significant background and poor compatibility with other types of fluorescence microscopy, where background from diffusing molecules cannot be distinguished for the signal of the bound species. On the other hand, the approaches based on fluorophore-target recognition frequently use environment-sensitive dyes which change their emission intensity (fluorogenic) or colour (chromogenic) in response to the binding event. Indeed, this is the case of Nile red-membrane, fluorogen-FAST, fluorogen-HaloTag or fluorogen-aptamer couples. Environment-sensitive dyes as fluorogenic and chromogenic probes to study biomolecular interactions have been previously reviewed.^61^ Here, we will mention the main classes of those probes, which are used to design probes for reversible labelling (Fig. 1C). First, solvatochromic dyes, operating by excited-state intramolecular charge transfer (ICT), change their color in response to polarity. Because these dyes also exhibit twisted intramolecular charge transfer (TICT),^62^ they usually light up on change in the environment from a polar aqueous to an apolar target. A typical example is Nile red, which is particularly suitable for reversible labelling of biomembranes, because of a strong change in the environment from polar water to highly apolar lipid membranes. It also provides information about local lipid organization (order) by change in its emission color. Second, restriction of intramolecular rotation (RIR) in fluorescent molecular rotors: they trigger their emission in highly viscous and rigid environments.^63^ Typical examples are the BODIPY^64^ rotor, ThT,^65^ and GFP derived fluorophores which are suitable for lighting up lipid membranes, protein aggregates (amyloid fibres) and RNA aptamers. They can also serve as probes of local viscosity using fluorescence lifetime imaging microscopy (FLIM).^64^ Then, ground-state isomerization, in particular spirolactone (or spirolactam) ring opening (SPIRO), is a highly popular mechanism to achieve fluorogenic light up on reversible binding, with key examples of rhodamine^11,23,66,67^ and silicon rhodamine^67–69^ derivatives. They are particularly suitable to light up engineered protein tags and RNA aptamers. Probes exploiting aggregation-caused quenching (ACQ) based on intramolecular dimerization, which include dye–quencher and dye–dye dimers, are another rapidly expanding approach. It is particularly suitable for lighting up RNA aptamers and biomembranes. All these types of dyes will be described in the review in relation to low-affinity labelling strategies. Their unique feature is that they not only allow imaging of the target, but also sensing its local properties.

All these methods of transient labelling have common goals and major challenges in the field of labelling and imaging. The first one is high specificity with minimal off target labelling, which is essential to obtain signals exclusively for the target of interest in the complex biological environment. Second is tuneable affinity, which allows optimal reversible labelling with appropriate binding–unbinding kinetics, essential for super-resolution and long-term imaging. In SMLM this kinetics should be fast enough to ensure rapid image recording but slow enough to provide sufficient photon count. Third is high brightness that is crucial in achieving high temporal and spatial resolution in fluorescence microscopy. Indeed, the number of collected photons is directly linked to the localization precision of the point emitter (see the next section), while the higher photon count rate allows faster image recording. The fourth challenge, strongly linked to the third one, is minimized background in order to obtain better image contrast and a higher signal to noise ratio. Fifth is minimized photobleaching and phototoxicity, because they enable long-term image recording without loss of the signal and impact on cell viability. Sixth is fluorogenic and/or chromogenic response to the binding event, which is important for achieving low background, super-resolution imaging and local environment sensing. Seventh is atom economy, which includes the molecular weight of the labelling agents used, the length of the linker if applicable, *etc.* It is especially crucial in super-resolution imaging, where the label should be smaller than the achievable resolution in order to minimize the linkage error (distance between the fluorophore and the target). Eighth is compatibility of the labelling method with live cell samples. It is mainly defined by cell permeability of the designed probes. Overall achieving high spatial and temporal resolution is a challenge that requires optimal combination of most of the mentioned factors. Finally, the labelling method should be simple, fast and cost-effective in order to be broadly used by the research community.

## 3. Fluorescence imaging and low affinity labelling

### 3.1 Super-resolution imaging

Fluorescence microscopy has become an invaluable tool for cell biology as it enables the investigation of cellular architecture and function across a wide range of spatial and temporal scales. Numerous labelling strategies allow the selective attachment of fluorophores or genetically encoded reporters to targets of interest in both fixed and living specimens. Because it is inherently non-invasive and compatible with time-lapse acquisition, far-field fluorescence microscopy has become a method of choice for monitoring biological processes over space and time. In its conventional form, however, optical microscopy is constrained by diffraction of light, which limits its spatial resolution to ∼200 nm laterally and ∼500 nm axially.

This barrier was overcome by the advent of super-resolution microscopy, which provides sub-diffraction imaging, opening the era of nanoscopy.^18–20^ Both scanning and wide-field implementations have been developed. Scanning approaches, including STimulated Emission Depletion microscopy (STED),^70,71^ Structured Illumination Microscopy (SIM),^72,73^ minimal photon flux imaging (MINFLUX, based on single molecule localization using a scanning approach),^74^ and 4Pi-microscopy,^75^ can provide outstanding resolution and optical sectioning, but they often rely on sophisticated instrumentation and stringent optical alignment. In contrast, widefield nanoscopy^76^ has become widely popular because it is based on detecting and localizing single emitters (Single Molecule Localization Microscopy (SMLM)). It relies on the controlled activation of only a small subset of fluorescent molecules at a time, allowing their positions to be determined with nanometer-scale precision. By repeating this process thousands of times and combining the localized positions of individual fluorophores, a high-resolution image of the sample is reconstructed. In SMLM, the diffraction-limited image of each isolated fluorophore is fitted with a model of its point spread function (typically a two-dimensional Gaussian), thereby achieving a localization precision that improves with the square root of the number of collected photons (*i.e.* the localization uncertainty scales as 
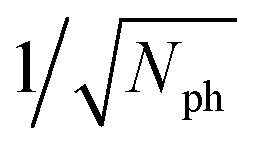
, Fig. 2A).^19,20,77^ This method can be implemented on conventional epifluorescence microscopes equipped with highly sensitive modern cameras.

**Fig. 2:**
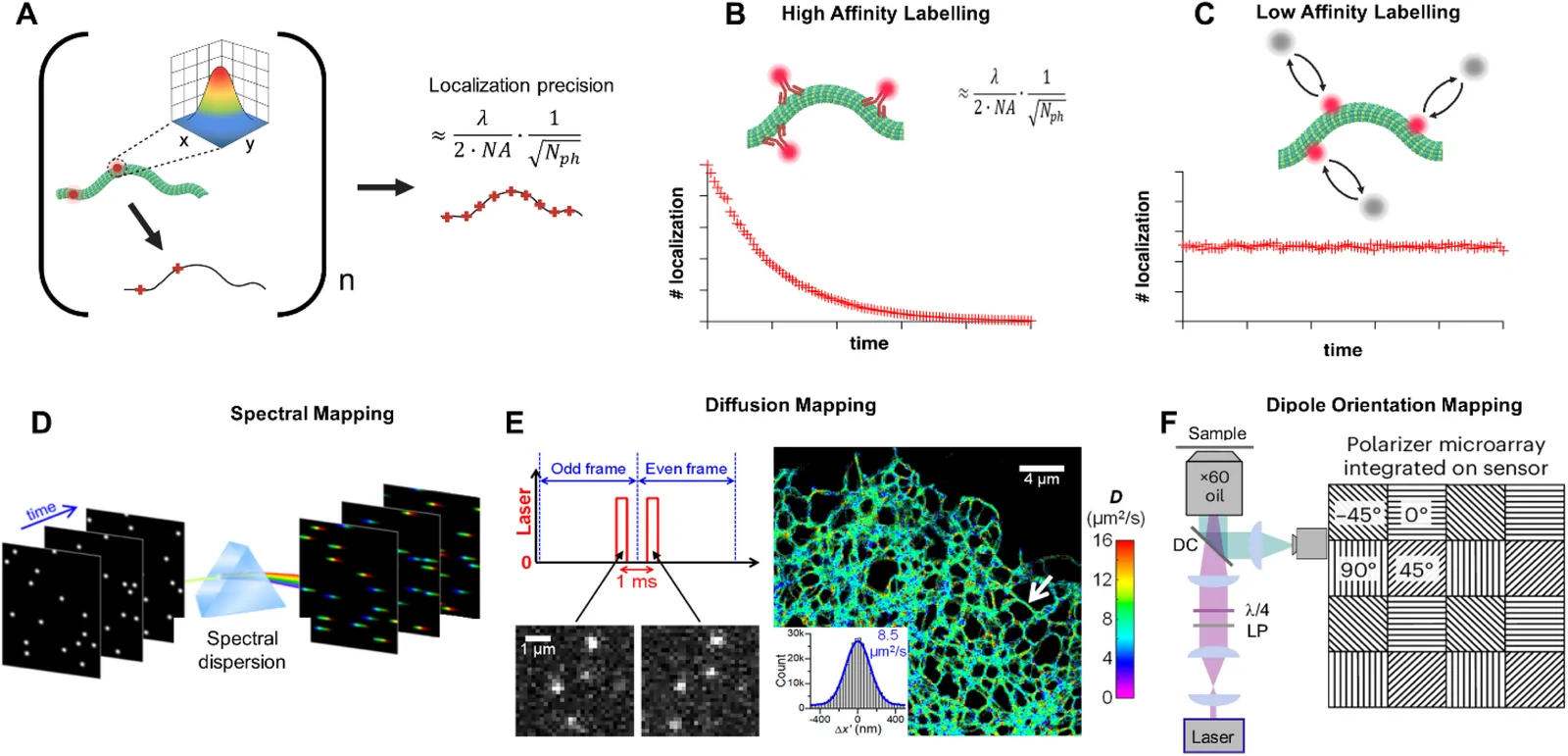
(A) Principles of single-molecule localization miscropy. High (B) and low (C) affinity labelling of biological targets. (D) Spectrally resolved PAINT imaging. Reproduced from ref. 87 with permission from ACS Publications, copyright 2018. Other dimensions of PAINT imaging include mapping of molecular diffusion (E)^41^ and orientation (F).^42^ Reproduced from ref. 41 with permission from ACS Publications, copyright 2020 for (E) and from ref. 42 with permission from Springer Nature, copyright 2024 for (F).

Despite being extremely powerful in resolving sub-diffraction structures, SMLM is intrinsically coupled to fluorophore development or the use of specific buffers as it relies on temporally separating sparse subsets of emitters, through photoswitching (*e.g.*, Stochastic Optical Reconstruction Microscopy (STORM)^77,78^ and Ground-State Depletion microscopy followed by Individual Molecule return (GSDIM))^79^ or photoactivation (*e.g.*, PALM),^19^ followed by precise localization and computational reconstruction. Although these methods can achieve very high spatial resolution, their performance is intrinsically constrained by fluorophore photophysics and by cumulative photobleaching, which can limit acquisition duration and complicate long-term imaging as the pool of active emitters is progressively depleted (Fig. 2B).

In this context, low-affinity exchangeable probes have been used to implement Point Accumulation for Imaging in Nanoscale Topography (PAINT).^30^ The PAINT technique is remarkable because the required blinking does not arise from intrinsic photoswitching, but from the transient binding of fluorescent probes. Therefore, low-affinity exchangeable probes elegantly address two persistent limitations of previously developed approaches (*e.g.* PALM or dSTORM). First, PAINT does not strictly require photoswitchable probes, which provides new opportunities in the probe-design. Nevertheless, fluorogenic behaviour (*i.e.* fluorescence turn-on upon binding or a strong increase in the quantum yield) remains highly desirable to maximize the signal-to-background ratio. A second key benefit is its robustness to photobleaching: since probes are not permanently immobilized on the target, individual dye molecules can bleach without impacting the signal at the labelling site because they are continuously replaced by intact probes from solution (Fig. 2C). This notion of a “quasi-infinite” dye reservoir enables acquisitions over tens of minutes, whereas permanently bound fluorophores often suffer from much faster cumulative signal loss. In addition, PAINT is highly modular in terms of its binding moiety and should be viewed not as a single technique, but as a family of variants. They can be classified based on the type of reversible binding, which is based either on ligand recognition with the most important example being DNA-PAINT^31,80^ or direct dye recognition with the primary example being PAINT of biomembranes using Nile red. The remarkable modularity of PAINT gave birth to many variants of the PAINT method beyond classical PAINT and DNA-PAINT: glyco-PAINT,^60^ peptide-PAINT,^56,81^ MotorPAINT,^82^ uPAINT,^83^ iPAINT,^84^*etc.* This modularity provides considerable freedom to tune affinity, selectivity, exchange kinetics, and compatibility with either fixed or living samples. Importantly, PAINT also offers exceptionally high resolution potential: in SMLM, resolution is determined using localization precision and the sampling density of independent events; by maximizing the number of binding events and carefully controlling their kinetics, PAINT can reach outstanding resolution, down to the Ångström scale.^22^ While such performance also demands exceptional mechanical and thermal stability as well as drift correction, PAINT is capable of providing the required oversampling. Importantly, PAINT naturally supports repeatable multiplexing: because labelling is reversible, imaging can be performed iteratively by acquiring one target, washing, and then introducing a different probe under similar optical conditions. With the help of orthogonal oligonucleotide sets, DNA-PAINT enables nearly unlimited sequential imaging cycles.^32,50,85^ An additional dimension in multiplexing is provided by exploiting exchange kinetics as a barcode, as it was shown for both DNA-PAINT^50^ and FAST protein PAINT.^86^

Additional dimensions in PAINT imaging can be provided by recording spectroscopic parameters of fluorescence. Thus, Lee and coworkers^39^ and Xu and coworkers^40^ introduced spectrally resolved PAINT (SR-PAINT), which provides both high resolution and spectral information at the single-molecule level. In this approach, a part of the camera is dedicated to single-molecule localization, while another part is used to record the spectrum (Fig. 2D). In practice, the fluorescence of each molecule is split between a non-dispersed channel, which is used for localization, and a dispersed channel, which uses a prism or diffraction grating to extract the full emission spectrum. Therefore, the emission maxima of every localized molecule become an additional coordinate in the super-resolved image. SR-PAINT is particularly suitable for solvatochromic dyes, like Nile red, which enabled nanoscale mapping of hydrophobicity and lipid organization of cell membranes.^39,40^ Further dimensions in the PAINT method were reached by recording molecular diffusion (Fig. 2E)^41^ and orientation (Figure 2F),^42^ which provided important information about biophysical properties of cell membranes. Diffusivity mapping (Fig. 2E) relies on single-molecule displacement measurements. Pairs of short stroboscopic excitation pulses, typically separated by a delay of ∼1 ms, are applied across consecutive camera frames. The displacements of the probe molecules between the two pulses are accumulated with nanoscale resolution and fitted with a two-dimensional random-walk model to yield local diffusion coefficients. In contrast, for orientation imaging (Fig. 2F), the fluorescence of each molecule is analyzed in several polarization channels. For example, a polarization-sensitive camera with pixel-level polarizers at 0°, 45°, 90° and 135° can be used to estimate the in-plane orientation and rotational freedom of the emission dipole for every localization, with minimal modification to a standard wide-field microscope. As the spectrum, mobility and orientation of a transiently bound solvatochromic dye such as Nile red depend on its immediate surroundings, these parameters provide information on local polarity, lipid packing and membrane order at the nanoscale.

Beyond these strengths, PAINT displays specific experimental constraints. A primary consideration is background management: a fraction of unbound probes is necessarily present in the observation volume. Fluorogenic probe design, excitation-volume restriction (Total Internal Reflection Fluorescence (TIRF) or Highly Inclined and Laminated Optical (HILO) microscopy), and careful concentration optimization are therefore essential. A second consideration is the need to operate in an appropriate kinetic regime: if the binding affinity is too strong, events last too long and overlap in space and time (too high a density preventing single molecule localization); if the binding affinity is too low, events become too rare (slow acquisition) or too short (insufficient photons acquired). In practice, the “optimal” affinity is the one that delivers an event rate compatible with camera speed and the photon budget while maintaining low average occupancy per target. PAINT demonstrates how low-affinity, dynamic, exchangeable labelling can become not a drawback, but a powerful approach to achieve super-resolution imaging.

### 3.2 Long-term imaging

Long-term conventional fluorescence imaging is essential for capturing biological processes that span over minutes to hours: *e.g.* cell migration, membrane trafficking, cytoskeletal remodelling, organelle dynamics, macromolecular assembly/disassembly, and cellular responses. In its standard implementation, covalent attachment of an organic fluorophore remains the major method for time-lapse imaging, because it is straightforward and provides high specificity. However, as mentioned above it comes with a fundamental limitation due to photobleaching. The latter causes an irreversible loss of the signal that can prevent continuous observation of the same labelled structures over long periods. In addition, the photobleaching mechanism is associated with production of reactive oxygen species, which induces phototoxicity and may impact the biological target, sometimes well before obvious morphological damage appears.^88,89^

An elegant approach to extend observation time is to replace permanent labelling with low affinity probes *via* reversible binding. Instead of a fluorophore that stays attached until it bleaches, the label is continuously supplied from the surrounding solution: a probe binds transiently, fluoresces, may bleach, then dissociates and is replaced by an intact molecule from the surrounding infinite reservoir. In the steady state, the signal becomes less constrained by the characteristic bleaching time of individual dyes and depends on the binding equilibrium and probe availability, which can maintain contrast over long acquisition time.^90^

In recent work, a common strategy for prolonged live-cell fluorescence imaging is to decouple labeling from permanent dye consumption by using fluorogenic ligands that bind reversibly from a reservoir, so that photobleached molecules are continuously replaced. Early fluorogen-activating proteins (FAPs) based on scFvs established the concept that a dark dye can become orders of magnitude brighter only when bound, enabling wash-free imaging.^91^ Based on this concept, the FAST/Y-FAST method was developed by Gautier and co-workers, providing a small (∼14 kDa) genetically encoded tag that forms a bright complex with cell-permeable fluorophores and dissociates rapidly making labelling fully reversible and tuneable in cells.^44^ To further improve this approach, they further demonstrated multicolour FAST imaging and dynamic colour switching driven by probe exchange allowing multiplexing and cross-correlation analysis,^86^ while engineered FAST variants (iFAST/td-iFAST) further improved brightness and binding performance for routine microscopy workflows.^92^ In bacterial communities, where GFP-like proteins can fail because chromophore maturation requires molecular oxygen, FAST–fluorogen labelling maintained quantitative signals over many hours and allowed tracking of biofilm growth dynamics in microenvironments displaying low oxygen concentration.^93^ Chemogenetic tags with probe exchange (CTPEs) were also developed: these tags use dyes that reversibly occupy hydrophobic pockets to provide fluorogenic signals and they can be renewed from solution to sustain longer imaging sessions.^94^ Two complementary approaches based on HaloTag technology now enable dye renewal. Retro-engineered HaloTags can be used to improve the turnover of fluorogenic substrates, supporting prolonged time-lapse imaging.^95^ Fully non-covalent, exchangeable HaloTag ligands (xHTLs) permit transient binding, enabling bleached rhodamines to be replaced and reducing photobleaching during long acquisitions; this also allows PAINT-like readouts to be obtained when desired.^33^ Finally, reducing fluorogen affinity can itself be engineered as an advantage. Azetidinyl Malachite Green (Aze-MG), for example, was designed to be brighter while exhibiting weaker binding to MG-FAP. This enables a buffering strategy, whereby the intact fluorogen continuously swaps in and out, stabilizing fluorescence over long recordings.^96^

Low affinity exchangeable probes also found applications in scanning-based high resolution approaches by continuously replacing photobleached fluorophores from the medium reservoir.^97^ In STED, fluorogenic labels that reversibly bind their targets provide a stable signal over long (conceptually unlimited) acquisition times, enabling whole-cell, 3D, multicolour live-cell imaging.^98^ For plasma membranes, the exchangeable Nile-red derivative NR4A supports minutes-long STED acquisition time without signal loss while simultaneously reporting lipid packing, allowing real-time 3D-STED of blebbing/fusion and STED measurements allowing characterization of diffusion and packing.^29^ By combining multiple transient interactions (exchangeable HaloTag ligands, weak DNA hybridization, *etc.*), multitarget STED is extended to six sequential targets in single cells, with reduced photobleaching and high-fidelity 3D/multicolour live-cell acquisitions.^99^ Exchangeable HaloTag ligands (xHTLs) generalize reversible protein tagging across colours, reduce photobleaching in STED, and also enable PAINT/MINFLUX dual-colour imaging.^33^ Retro-engineered HaloTag has been produced and displays seconds-to-minutes ligand turnover, provides 3 to 5 times higher photostability, and allows controllable single-molecule labelling density over extended time windows for SIM imaging of organelle dynamics.^95^ Exchangeable probes such as APBD enable wash-free time-lapse SIM and were used to track necrosis-associated mitochondrial remodelling and intracellular water heterogeneity in a kidney injury/repair context,^34^ while tuned fluorogen activated protein binding (Aze-MG) supports an exchange-based “buffering” strategy for stable dynamic SIM.^96^ Overall, these studies evidenced that transient and low-affinity labelling can significantly prolong high-resolution experiments by reducing photobleaching while maintaining the biological system in its native state, provided kinetics, concentration, and background are carefully controlled.^36,100,101^

## 4. Probes for low-affinity labelling

As explained in the section above, we could classify all reversible probes into those based on fluorophore and ligand recognition. This classification is simple and could be applied to most examples, except those making use of both the fluorophore and ligand in the recognition processes.

### 4.1 Probes based on fluorophore recognition

#### 4.1.1. Probes for biological membranes

Biological membranes were probably the primary target where the power of low affinity labelling was shown with a seminal report by Hochstrasser and co-workers in 2006.^30^ Indeed, using solvatochromic and fluorogenic dye Nile red, reversible binding was exploited to achieve dye immobilization with fluorescence turn on and further super-resolution imaging of model lipid membranes (Fig. 3A and B). The driving force here is the fluorophore-membrane recognition, which stems from the lipophilic nature of Nile red and relatively simple polycyclic structure that allows its easy accommodation within the lipid bilayer. On the other hand, fluorescence light-up of this dye stems from solvatochromism of Nile red and the change in the environment from highly polar water to the apolar lipid membrane.^61^ Further studies exploited sensitivity to polarity of Nile red to introduce spectrally resolved PAINT (SR-PAINT),^87^ which provides spectral information for single molecules bound to the membrane. SR-PAINT provided access to lipid order imaging at the nanoscale,^39,40^ because the emission maximum of Nile red depends on the lipid organization of biomembranes.^102^ Thus, SR-PAINT revealed fine differences between the highly ordered plasma membrane and much less ordered endoplasmic reticulum and mitochondria (Fig. 3C and D).^40^ However, the limitation of this approach for plasma membranes is non-specific accumulation of Nile red inside the cells. Our early studies suggested that Nile red could be targeted specifically to plasma membranes by addition of a membrane anchor; however, the obtained probe NR12S was designed to have high affinity to the membrane.^102^ Therefore, our group introduced a reversible membrane anchor, which enabled Nile red-based probe NR4A to target reversibly the plasma membrane with minimal cell penetration.^43^ In contrast to high affinity probes (NR12S and its analogue NR12A), which showed very poor blinking behaviour due to nearly irreversible membrane binding (Fig. 3E and F), NR4A with low affinity to the membrane was perfectly compatible with PAINT imaging (Fig. 3E and G). Remarkably, the membrane anchor also improved single-molecule brightness, provided faster ON–OFF switching kinetics (Fig. 3E) and provided higher signal-to-noise ratios, which highlights the importance of the rational design of low-affinity probes. Beyond PAINT imaging, the NR4A probe enabled long-term STED imaging of cells with minimal cytotoxicity^29^ and minimized artefacts related to dye photodegradation that leads to photobluing.^103^ More recently, we replaced Nile red with a fluorogenic dimer of cyanine dyes, which boosted the brightness and decreased diffusion of the probe in the membrane, significantly improving the spatial (3-fold down to 31 nm along *z*-axis) and temporal resolution of PAINT of plasma membranes.^37^ The use of reversible binding to the cell membrane is not limited to the plasma membrane. Xu and co-workers also proposed the Nile red probe for PAINT imaging of intracellular structures, such as the ER and mitochondria.^40^ However, the limitation of PAINT here is the limited access to the dye pool, because of the limited internalization rate of Nile red inside the cells. In another study, a reversible membrane binder was recently designed based on APBD dye, which enabled long-term structured illumination imaging (SIM) of mitochondria and the ER.^104^ This study revealed interactions of those two organelles and nanoscale structural changes of their membranes during necrosis. The use of SIM is probably more appropriate for intracellular imaging of exchangeable probes, as it does not require fast dye exchange with a bulk medium.

**Fig. 3:**
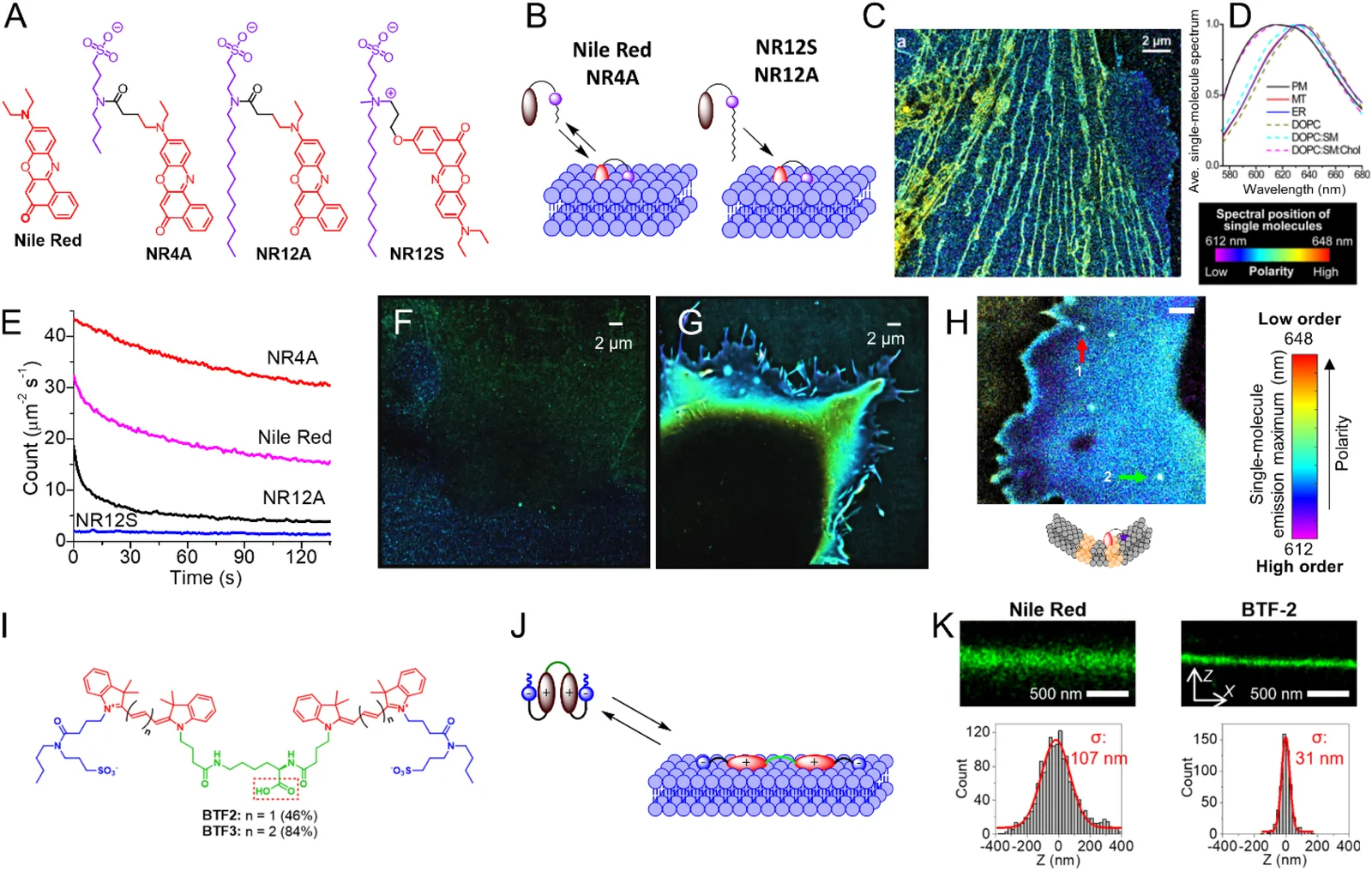
PAINT probes for biomembranes. (A) Chemical structure of membrane probes based on Nile red. (B) Scheme presenting reversible and irreversible labelling of lipid membranes. (C) Spectrally resolved PAINT imaging of cell membranes of live PtK2 cells using Nile red. Each detected single molecule is colour-coded according to its spectral mean [colour bar in D]. (D) Averaged spectra of single Nile red molecules from different nanoscale regions in live cells at the plasma membrane (PM), mitochondria (MT), and ER, compared to that at model supported lipid bilayers of different compositions. Reproduced from ref. 40 with permission from ACS Publications, copyright 2017. (E) Time-dependent counts of single molecules detected per µm^2^ per second for the four Nile red-derived probes. 3D PAINT imaging of COS-7 cells with (F) NR12S (20 nM) and (G) NR4A (10 nM). (H) Nanoscopic mapping of lipid order of the plasma membrane of live COS-7 cells using SR-PAINT with the NR4A probe. Colour represents single-molecule spectral mean (see the colour scale bar). (Below) Scheme showing that membrane curvature may impact the lipid order imaged using the probe. Reproduced from ref. 43 with permission from Wiley, copyright 2019. (I) Fluorogenic dimers (BTF2 and BTF3) based on cyanine dyes as bright PAINT probes and the scheme of their light-up on binding to the membrane surface (J). (K) Typical vertical cross-sections of the 3D-PAINT data of the two probes at the plasma membrane (top) and the spread in *Z* values for the local flat regions. Gaussian fits (red curves) give standard deviations of 107 and 31 nm, respectively. Reproduced from ref. 37 with permission from ACS Publications, copyright 2022.

#### 4.1.2. Engineered protein tags

Genetically encoded protein tags are very popular tools for covalent labelling of proteins (*e.g.* HaloTag, SNAP-tag, Clip-Tag, *etc.*).^105^ Recently, the ligand for labelling of HaloTag was re-designed in order to prevent it from covalent coupling with HaloTag. The developed ligands (xHTLs) bearing a rhodamine-derived (JF-635) dye were able to reversibly bind HaloTag, allowing low affinity labelling of proteins bearing HaloTag for both STED and PAINT imaging.^33^ One should note that even though this method exploits ligand recognition, we mention it in this section, because it is broadly accepted that part of the binding site of HaloTag recognizes the rhodamine-based fluorophores. Therefore, there is a significant effort to develop fluorogenic rhodamine based HaloTag ligands.^106–108^ The reversible nature of HaloTag labelling could significantly reduce photobleaching as compared to covalent HaloTag labelling in super-resolution STED imaging.^33^ Moreover, transient binding of fluorogenic xHTLs to HaloTag fusion proteins enabled PAINT and MINFLUX microscopy.^33^ Moreover, pairs of xHTLs and HaloTag mutants were developed that enabled dual-colour PAINT and STED microscopy. Further studies also showed that xHTLs can be applied for long-term imaging of proteins in cells with minimal impact of photobleaching and phototoxicity.^34^ Moreover, xHTLs were successfully combined with neural networks (DeepSTORM) to reconstruct super-resolution images from high-density imaging data, which allowed reduction of the acquisition time up to ∼25-fold.^109^ An intrinsically non-covalent approach to protein labelling is based on a protein tag FAST, where a binding site of the protein was engineered to reversibly bind a fluorogenic dye based on fluorophore analogues of a green fluorescent protein.^44^ The protein cavity of FAST allows the stabilization of the deprotonated state of the chromophore and favours its planar conformation, which leads to its fluorescence light up. Therefore, this method allowed labelling target proteins bearing the FAST tag without washing out the unbound fluorogenic dye. Moreover, systematic protein engineering yielded FAST proteins that light up fluorogenic dyes of different colours allowing multi-colour imaging of cellular protein targets.^110^ The FAST labelling approach found numerous applications in advanced microscopy,^110^ ranging from classical confocal microscopy to single molecule tracking and super-resolution microscopy. Thus, dynamic exchange of the fluorogen ensured ON/OFF switching behaviour of FAST tags, which enabled single-molecule localization microscopy with 28 nm resolution.^111^ Moreover, this tag approach enabled dynamic colour switching by exchange between green to red fluorogens within the FAST tag. It allowed imaging the FAST protein together with spectrally overlapping green and red fluorescent proteins using only two detection channels, which can push the limits of multiplexed imaging.^86^ Recently, protein tags were engineered from multidrug-resistant proteins to bind specifically rhodamine (Rho-Tag) and silicon rhodamine (SiR-Tag) dyes, which opens new avenues for reversible fluorescent labelling of biological systems *in vitro* and *in vivo* given the high brightness and tunability of the rhodamine dye family.^69^ Moreover, *de novo* design of proteins yielded orthogonal protein tags NovoTags of far-red, orange, and green fluorophores that enabled multiplexed imaging.^66^ These recent studies highlighted the vast possibilities for designing protein binders for small-molecule fluorophores, thus offering a powerful toolbox for reversible labelling of proteins, which will be particularly important for advanced imaging methods such as super-resolution, multiplexed and long-term imaging.

#### 4.1.3. Fluorogenic RNA aptamers

Similar to proteins, RNA aptamers can serve as biomolecules that can specifically bind a fluorogenic dye providing low-affinity labelling. Here, an engineered RNA aptamer presents a specific binding pocket that enables specific binding of the dye associated with fluorescence light up due to planarization and/or rigidification of the dye environment (Fig. 4A).^112^ The first identified fluorogenic dye that could be lit up using an aptamer was malachite green, reported by Tsien and co-workers.^113^ Later on, Jaffrey and co-workers introduced the Spinach aptamer paired with the DFHBI fluorogenic dye, whose structure is close to the structure of the GFP fluorophore, showing the first application for RNA imaging in live cells.^46^ Since then, a large number of aptamer/dye pairs were developed generally using the SELEX strategy, which includes broccoli/DFHBI,^114^ corn/DFHO,^115^ mango/TO-1,^116^ DNB/TMR-DN,^117^ o-Coral/Gemini,^118–120^ biRhoBAST-TMR_2_,^121^*etc.* While most fluorogenic dyes for light-up aptamers are based on molecular rotors, alternative approaches have been reported, such as using dye-quencher heterodimer TMR-DN^122^ (Fig. 4B) and self-quenched homodimers, like Gemini or TMR2 (ref. 121) (Fig. 4C). These approaches enabled imaging directly in live cells a variety of RNAs such as mEGFP (Mango, o-Coral, biRhoBAST, *etc.*),^116^ TNFα (biRhoBAST),^121^ β-actin mRNA (biRhoBAST),^121^ and RNA stress granules (biRhoBAST),^121^ as well as imaging RNA polymerase II transcription^115^ or tracking single molecules of mRNA.^121^ Moreover, these light-up aptamers can be engineered to introduce sensing modules to turn them into fluorescent sensors (Fig. 4D)^123^ for various analytes such as nucleic acids^124,125^ metabolites^126,127^ or even small ions such as fluoride (Fig. 4E and F).^128^ Such sensors are usually low-structured RNA sequences that require first binding to the analyte of interest to pre-fold and allow the binding of the corresponding dye to form a tertiary fluorescent complex (Fig. 4D). The exchangeable nature of such systems allows designing dynamic sensors that can respond in real time to a variation of concentration of the analyte of interest. More recently, combination with specially designed fluorogenic probes with aptamers of low affinity were proposed for super-resolution PAINT imaging of RNA in live-cells. Thus, Sunbul, Jäschke and co-workers introduced several pairs of RhoBAST aptamers with fluorogenic dyes based on dye/quencher heterodimers (Fig. 4B)^129^ or silicon rhodamine dimers (Figure 4C),^121^ with a low affinity in order to obtain a spontaneous blinking necessary for PAINT imaging. Recently, Sunbul and co-workers exploited spirolactone opening as a mechanism for fluorogenic response of rhodamine dyes to interaction with the RhoBAST aptamer.^67^ Such aptamer-based probes allow the highly specific detection of the aptamer of interest ensuring background-free and wash-free imaging conditions that allow imaging circular structures of RNA in the nuclei of live HEK293T cells and mRNA aggregates in fixed COS7 cells with resolution close to 80 nm by SMLM imaging (Fig. 4H and I).^67^ Although light-up aptamers are largely promising for RNA, their rational design for low-affinity labelling and PAINT imaging emerged only recently.

**Fig. 4:**
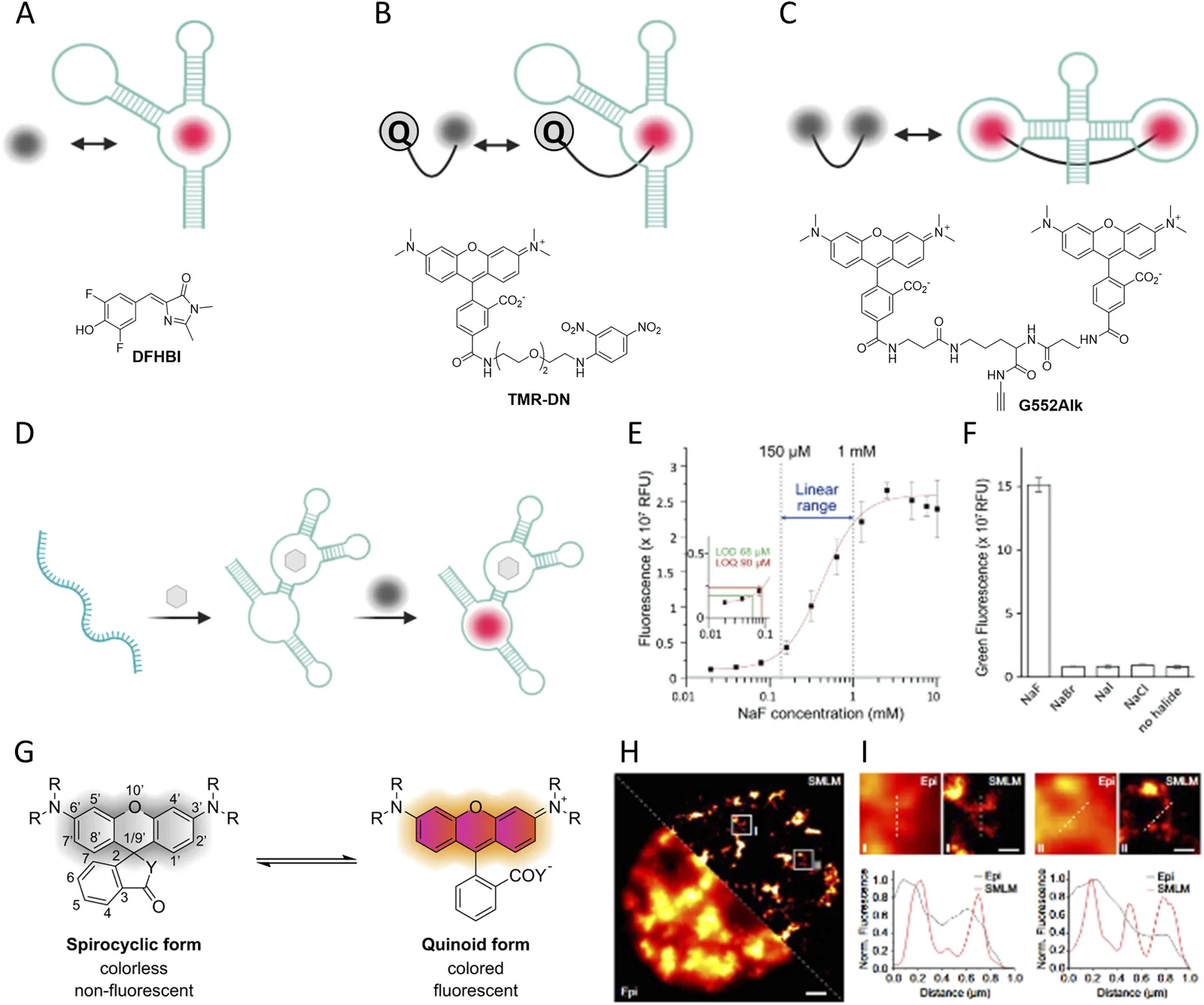
Fluorogenic aptamers. Concepts of aptamers paired with fluorogenic dyes based on (A) fluorogenic dye operating by a molecular rotor (environment rigidification) mechanism with DFHBI as an example,^46^ (B) dye-quencher heterodimers, such as TMR-DN^122^ and (C) self-quenched homodimeric dyes, such as G552Alk.^119^ (D) Fluorogenic aptamer-based sensors for fluoride detection using FluorMango. (E) Limit of detection (LOD), limit of quantification (LOQ), and linear range of fluoride detection. Values are the mean of seven (no NaF) or three (other NaF concentrations) independent experiments, and error bars correspond to ±1 standard deviation. LOD was derived from three times the standard deviation, and LOQ from ten times the standard deviation. (F) Specificity of FluorMango to a variety of sodium halides. Values are the mean of three independent experiments, and error bars correspond to ±1 standard deviation. Reproduced from ref. 128 with permission from Wiley, copyright 2023. (G) Scheme of the fluorescence light up of a rhodamine derivative induced by interaction with a RhoBAST aptamer. (H) Epifluorescence (Epi) images of fixed Cos7 cells expressing CGG99-FMR1-GFP-RhoBAST16 mRNA incubated with SpyRho (1 nM) for 30 min before imaging. (I) Top: close-ups of the regions indicated by the white frames; bottom: normalized intensity profiles along the white lines. Scale bars, 2 µm (H) and 500 nm (I). Reproduced from ref. 67 with permission from Springer Nature, copyright 2023.

#### 4.1.4. Endogenous biomolecules

Fluorogenic dyes could also provide reversible binding to endogenous biomolecules, naturally produced in cells, such as proteins and nucleic acids. This enables their labelling without genetic modification and further imaging with advanced microscopy. A classic example is reversible labelling of amyloid fibres with thioflavin T, which is a molecular rotor dye that lights up, because the binding to the sites of high environment rigidity leads to restriction of intramolecular rotation in the excited state.^65^ The exchangeable nature of this labelling enabled long-term STED super-resolution imaging of amyloid fibres down to 60–70 nm resolution with a low background and an increased photostability.^130^ The same transient labelling approach was further extended to a near-infrared fluorogenic curcumin dioxaborine derivative CRANAD-2, allowing both STED and SMLM imaging of amyloid fibres with 45–55 nm resolution and much higher photostability compared to the covalently attached ATTO-647N dye.^131^ One should also mention transient binders of DNA, which constitute a broad research field, beyond the scope of this work. However, we should mention here the approach of dye conjugation to Hoechst 33258,^132^ a dye exhibiting good specificity to double-stranded DNA. Thus, the conjugation of silicon-rhodamine with Hoechst 33258 yielded a fluorogenic probe for chromatin staining and STED imaging with resolution below 100 nm.^68^ The design was further extended to Janelia Fluor dyes, resulting in a toolbox of fluorogenic probes for PAINT super-resolution imaging of the *E. coli* nucleoid,^133^ revealing its proximity to the inner membrane and fine changes in its morphology under influence of transcription, translation and cell geometry.^134^

### 4.2 Probes based on ligand recognition

#### 4.2.1. DNA-PAINT

Reversible and highly specific hybridization of DNA is a powerful approach for low affinity labelling of DNA and proteins (Fig. 5A).^31,32^ This labelling approach gave rise to DNA-PAINT, a super-resolution microscopy technique first introduced by Jungmann, Tinnefeld and co-workers for DNA origami structures (Fig. 5B), achieving a resolution of 80 nm.^31^ The method was subsequently adapted by Jungmann and co-workers for cellular imaging, enabling a resolution of 10 nm for DNA origami structures and 100 nm for fixed cells by 2014.^32^ To allow visualization of biological structures at the nanometer scale, by taking advantage of programmability, renewability, and orthogonal multiplexing of DNA nanotechnology,^135^ DNA-PAINT uses two single stranded DNA (ssDNA) strands to label target molecules in a sample. The ssDNA attached to the target of interest (*e.g.* antibody) is called the docking strand and the complementary ssDNA bearing a fluorophore and freely diffusing in the environment is called the imager strand. The initial DNA-PAINT imager strands reported by Jungmann *et al.* were based on a 10 nucleotide sequence 3′ modified with Alexa655 or Cy3B.^31^

**Fig. 5:**
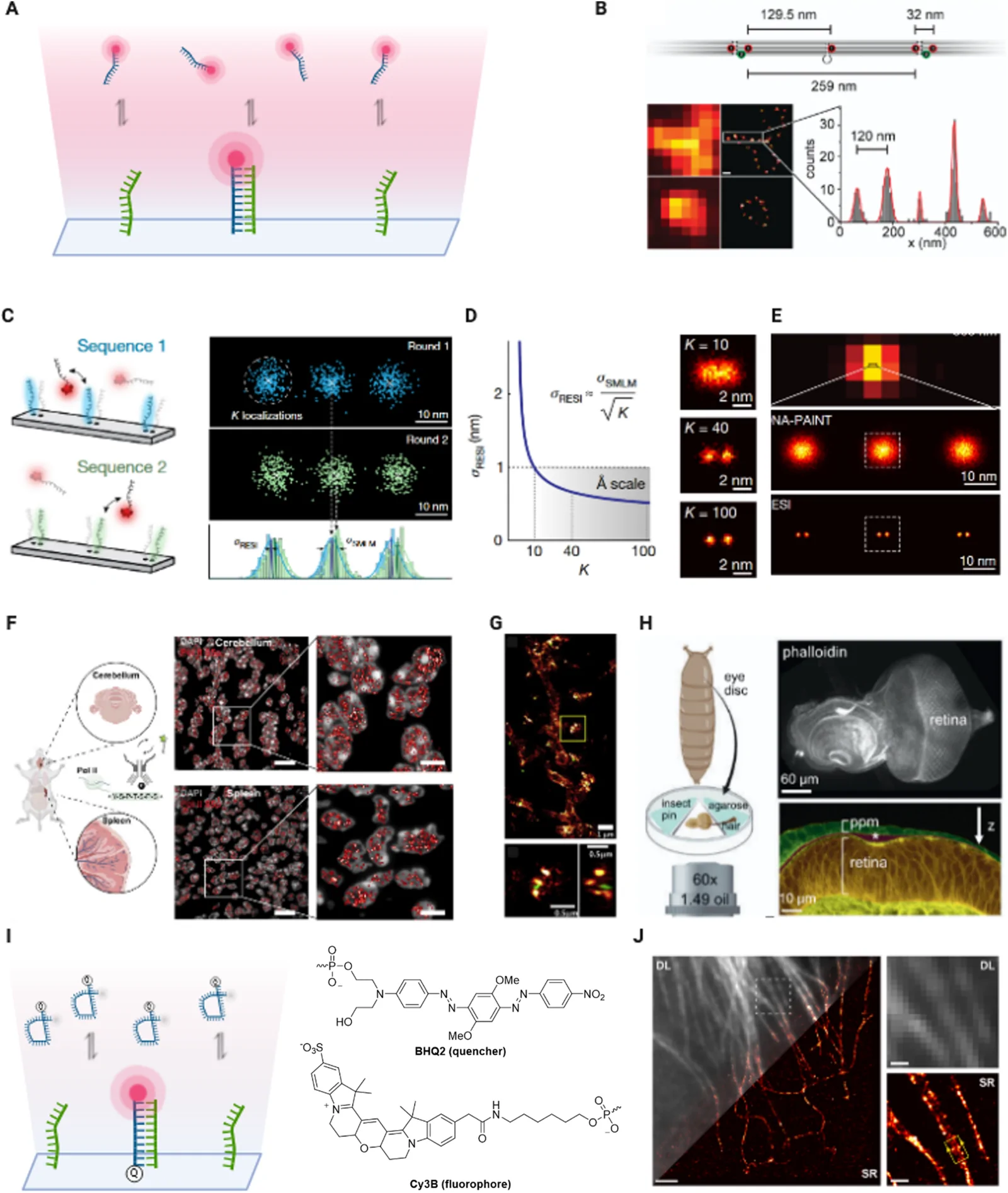
DNA-PAINT. (A and B) Diffraction-limited TIRF and super-resolved DNA-PAINT images of triple labelled oligomers with connection strands every 129.5 and 32 nm. The 1D histogram of a region of interest containing several attachment sites (length scale, 120 nm). Reproduced from ref. 31 with permission from ACS Publications, copyright 2010. (C) Principle of RESI: performing repeated sequential acquisition with orthogonal DNA sequences (blue and green) allows statistics of localization from SMLM to be enhanced. (D) Improvement of resolution with the number of cumulated sequences of acquisition. (E) Comparison of resolution between diffraction limited widefield, DNA-PAINT and RESI. (C–E) Reproduced from ref. 22 with permission from Springer Nature, copyright 2023. (F) Tissue blocks of the mouse cerebellum and spleen processed by cryosectioning and stained for Pol II S5p prior to imaging. Top: the tkPAINT image of a mouse cerebellum region and a zoom-in of the highlighted area. Bottom: the tkPAINT image of a mouse spleen region and a zoom-in of the highlighted area. Reproduced from ref. 147 with permission from NAS, copyright 2025. (G) Live-cell 3D-DNA-PAINT image of endogenous GluA1-AMPAR in live hippocampal neurons. Images were recorded with a 1 nM R1_8nt-LD655 imager. Top: the reconstructed DNA-PAINT image of GluA1-AMPARs (red hot) and Homer1 (green). Bottom: magnified view of the highlighted spine of the neuron in the yellow square in the lateral (*x*–*y*, left) and vertical projection (*y*–*z*, right). Reproduced from ref. 137 with permission from Cell Press, copyright 2023. (H) DNA-PAINT imaging of *Drosophila* retinal epithelium on an SDC-OPR microscope with up to 9 µm penetration depth. Left: schematic of the 3rd instar *Drosophila* eye disc and sample mounting for SDC-OPR imaging, described in Methods. Right: the phalloidin-stained eye disc (top). The peripodial membrane (ppm), luminal space (*), and retinal epithelium (retina) are highlighted with green, magenta, and orange masks, respectively (bottom). Reproduced from ref. 141 with permission from Springer Nature, copyright 2025. (I) Principle of the fluorogenic imager strand with a quencher group with the chemical structure of the most common fluorophore/quencher dyad. (J) Diffraction-limited (DL) images alongside super-resolution (SR) images of the microtubule network in a HeLa cell (left). Zoom-in diffraction-limited and super-resolved representations (right) of the highlighted area in the overview on the left demonstrate the increase in resolution. Imaging conditions are a 500 nM donor imager, 14 ms integration time, and 2000 frames, resulting in a total imaging time of 28 s. Scale bars, 2 µm (overview in J) and 500 nm (zoom-in images in J). Reproduced from ref. 153 with permission from ACS Publications, copyright 2017.

Similar to the classical PAINT method, DNA-PAINT exploits the transient binding (hybridization) of the imager strand on the docking strand to generate fluorescent signals by immobilization of the probes (Fig. 5A). As the exposure time of the camera in a typical DNA-PAINT protocol is relatively long (200–300 ms per frame),^3^ rapidly diffusing probes are detected as a background, allowing localization of only species bound at a specific location during that period of time, generating apparent blinking events. For this reason, the original DNA-PAINT approaches are limited by high background noise, slow acquisition rates, and poor compatibility with live-cell imaging. On the other hand, DNA-PAINT benefits from a highly specific detection and its ability to detect selectively single targets allowing quantitative imaging (qPAINT).^136,137^ In addition, as the DNA hybridization is a robust repeatable process, it allows extended acquisition time and makes the approach less sensitive to photobleaching as free imager probes in the solution replenish photobleached ones.

Classically, the resolution of SMLM techniques (*σ*_SMLM_) is determined by the number of photons collected by events: 
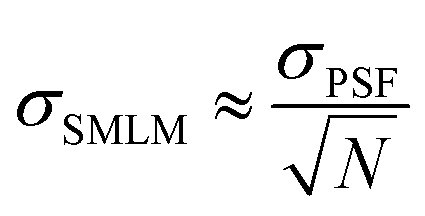
 where *σ*_PSF_ is the standard deviation of the point spread function and *N* is the number of photons collected. In the case of DNA-PAINT, the tolerance to photobleaching and possibility to exchange the imagers allow a large number of binding events to be accumulated and multiple acquisition sequences to be performed to increase the statistics of localization. This approach, named resolution enhancement by sequential imaging (RESI) by Jungmann and co-workers, is reported to enable resolution down to the Ångström scale (7 Å) with 
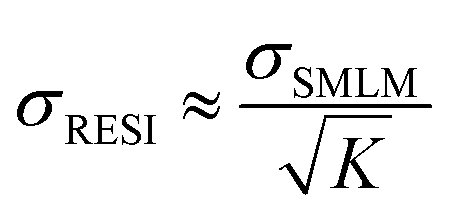
 where *K* is the number of localizations determined using the target (Fig. 5C–E).^22^ Such resolution allowed the authors to resolve the spatial organization and clustering of two antibodies (rituximab and obinutuzumab) on CD20 proteins on fixed CHO-K1 cells and to correlate antibody–antigen arrangement with therapeutic function in immunotherapies.^138^ This approach was further applied to visualization of individual sugars within glycans on the surface of fixed cells, which enabled imaging of the glycocalyx with a spatial resolution down to 9 Å.^139^

Over the past decade, many developments in DNA-PAINT have been reported in the literature to overcome most of the initial limitations of this approach following several independent directions.

In terms of targets, the first DNA-PAINT experiments were reported on model structures based on DNA origamis.^31^ However, it has been rapidly extended to biological targets at the cellular level. Primarily it was proposed in combination with antibodies conjugated to a docking oligonucleotide.^32^ The modular nature of DNA-PAINT allowed the use of an impressive number of other biomolecular vectors for target-specific low-affinity labelling, including nanobodies,^140,141^ RNA aptamers^142^ and affimers,^143^ enabling the visualization of a wide range of biologically relevant targets.^144^ A variety of classical bioconjugation techniques have been used to link the docking strand to the targeting protein, which includes peptide coupling,^145^ click chemistry and thiol–maleimide coupling.^136^ Finally, genetically encoded docking strands have been reported as an alternative targeting strategy that uses hybridization as the only recognition mechanism.^145,146^

DNA-PAINT has already been established as a toolbox with numerous biological applications. As a selected example, Jungmann *et al.* reported super-resolution imaging of proteins located in various organelles, of fixed cells such as β-tubulin (microtubules), COX IV (mitochondria), TGN46 (Golgi apparatus) and PMP70 (peroxisomes).^32^ Several studies extended DNA-PAINT to more challenging samples. Thus, Stein *et al.* coupled 3D DNA-PAINT imaging with ultrathin cryosectioning to image Pol II S5p in spleen and cerebellum tissues at 3 nm *x*–*y* resolution in 150 nm sections with 8 nm *z* resolution (Fig. 5F).^147^ They also extended the method to multicolour imaging by targeting laminA/C (lamina) and SC35 (nuclear speckles) by integrating exchange-PAINT multiplexing. In another study, Youn *et al.* adapted DNA-PAINT to live cell surface imaging.^137^ By incorporating up to 16 repeated binding motifs into the docking strand, the authors addressed the challenge of achieving rapid and specific imager-strand binding under physiological buffer conditions. This strategy enabled the mapping of AMPA receptors in cultured live neurons through the targeting of the GluA1 subunit and Homer1 protein at synaptic sites (Fig. 5G).

The high specificity of DNA strand hybridization allows multiplexed DNA-PAINT by the sequential use of exchangeable imager strands (exchange-PAINT) labelled with the same dye^32^ or by simultaneous acquisition using multicolour imaging.^148^ As DNA-PAINT does not rely specifically on fluorophore photophysical properties, a large variety of dyes are compatible and available for DNA-PAINT.^149^ They include widely used commercially available dyes such as cyanines, Atto, Alexa Fluor, Janelia Fluor or CF probes. Even though using multiple colours is the most common approach for multiplexing fluorescence microscopy acquisition, other properties are exploited in the literature to add additional channels such as lifetime, kinetic/frequency^50^ or combining right-handed DNA with its mirror-image analogues – left-handed DNA.^150^

Important efforts have been made to optimize and improve DNA sequences and imaging conditions to increase multiplexing possibilities and enhance acquisition speed. In this way, repeat-PAINT, which consists of repeating binding sequences on the docking strands, can reduce non-specific events by ∼10-fold, reduce the background by ∼5-fold and accelerate data acquisition by 5–10-fold without impacting the spatial resolution.^151^ DNA-PAINT-ERS, which involves repeating sequences on the docking strand, the use of ethylene carbonate in the imaging buffer, and the introduction of a spacer between the docking strand and the binding agent, has been reported to improve the kinetics of hybridization.^152^ These optimizations are mostly focused on increasing the turnover of imager strands to increase the kinetics of exchange but without reducing the specificity.

To reduce the inherent high background from non-bound imaging strands, several strategies have been introduced. One consists of using the FRET pair (FRET-PAINT) with a donor docking strand or a second acceptor dye that interacts with the docking-imaging strand complex (Fig. 5J).^153,154^ This method allows reduction of the background by avoiding detection of free fluorescent reporters and permits increasing the probe concentration, thus speeding up the image acquisition by several orders of magnitude.^153^ A similar approach has been reported using dark resonance energy transfer (DRET), where an intrinsically quenched energy donor can provide energy and light up the imager probe through energy transfer.^155^ Another strategy consists of developing fluorogenic imagers that are self-quenching in water and light-up upon binding on the docking strand.^156,157^ Thus, Chung *et al.* reported a fluorogenic imager displaying a 57× turn-on factor allowing a 26-fold increase in imaging speed by functionalizing an imaging strand with Cy3B (fluorescent probe) and BHQ2 (quencher) (Fig. 5I).^157^ Indeed, the reported fluorogenic system by reducing the background noise of DNA-PAINT enabled the use of 200-fold higher probe concentration (from 100 pM to 20 nM), which increased the blinking frequency from 0.2 blinks per µm per s to 4.3 blinks per µm per s, thus increasing the imaging speed 25.8-fold while maintaining the signal to noise ratio sufficient to perform precise localization. The authors used this fast acquisition DNA-PAINT to perform dual colour imaging of endoplasmic reticulum (protein Sec61β) and mitochondria (protein TOM20) in only 10 min under wide field illumination on fixed cells. Moreover, by using efficient FRET from ultrabright light-harvesting nanoparticles to the surface, we showed that single-molecule hybridization was accompanied by light up of the imager probe bearing the FRET acceptor, which led to nearly 100-fold signal amplification using ultralow power of a basic epi-fluorescence microscope and low background.^158^

The concept of DNA-PAINT makes it compatible with many advanced microscopy techniques. Thus, illumination techniques such as TIRF^159,160^ or HILO^32,149^ have been employed with DNA-PAINT, which could address its key limitation – high background generated by the unbound imagers. Other illumination techniques have been developed to increase the size of the field of view (FOV) such as Adaptable Scanning for Tunable Excitation Regions (ASTER)^161^ that increase the FOV from 40 × 40 µm^2^ to 200 × 200 µm^2^ by using fast scanning illumination and PRism-Illumination Microfluidics-Enhanced DNA-PAINT (PRIME-PAINT)^162^ that reached up to 520 µm by 520 µm FOV using a prism as a light waveguide and a microfluidic chip to supply DNA-PAINT buffers in an efficient way for multi-colour imaging. As a single molecule based technique, DNA-PAINT is also generally coupled to 3D-SMLM systems^32^ based on the engineering of the shape of the PSF to encode the axial position to directly image in three dimensions.^163,164^ Moreover, spinning disk microscopy was combined with angstrom-resolved RESI DNA-PAINT 3D imaging by optical sectioning.^141^ In this approach, Zaza *et al.* achieved 3D imaging of a range of biological targets using exchange-PAINT multiplexing, including nuclear pore complexes, mitochondria and microtubules with a spatial resolution going from 6 nm in the basal plane to 16 nm at 9 µm depths, on a large FOV of 53 × 53 µm^2^.^141^ This technique was successfully applied to image the retinal epithelium of a *Drosophila* eye disc (Fig. 5H). The transient labelling in DNA-PAINT was successfully applied for super-resolution optical fluctuation imaging (SOFI) of subcellular structures with minimized photobleaching.^165^ Later on, the SOFI methodology was applied in combination with exchangeable oligonucleotide labels to achieve 30–300-fold faster imaging of different cellular structures compared to conventional DNA-PAINT imaging.^166^ Recently, new advanced techniques have been combined with DNA-PAINT such as MINFLUX^167,168^ to avoid fluorophore bleaching issues^169^ and reach sub-nm resolution without using longer RESI sequences.^170^ In addition, DNA-PAINT was combined with advanced sample preparation techniques such as sample clearing^160^ or cryosectioning^147^ to enhance the imaging quality, especially for thick samples. Finally, dedicated computational methods were involved to assist experiments of increased complexity and accelerate the processing of the large volume of data generated by more and more high content DNA-PAINT acquisitions, mostly *via* machine learning and deep neural networks.^171,172^

Despite the high number of DNA-PAINT applications, few researchers reported live cell imaging with this approach that generally requires cell fixation and permeabilization, first due to the relatively long acquisition time required and the lack of cellular permeability of the imaging strands. Reported examples of live DNA-PAINT are limited to the cell surface topography or receptor sensing.^173,174^ Most of the LIVE-PAINT approaches replaced DNA strands by peptide-protein interactions (see below).^174,175^

While DNA-PAINT is widely used for cellular imaging, it is also reported for nanomaterial functional characterization, such as ligand availability on the nanoparticle surface,^176^ which could not be done by electron microscopy, or for biomarker detection and quantification in biological samples with qPAINT methods.^177,178^ DNA-PAINT also found other applications such as single-particle tracking, which allowed long-term recording due to replenishment of the probe on the particle surface.^179,180^

Overall, DNA-PAINT is a very powerful method based on exchangeable probes, is compatible with a large range of standard instrumentation and has become widely used for super resolution microscopy. While many advances and enhancements in DNA-PAINT have been reported in the past decade, some intrinsic limitations of this technique remain, which include long acquisition time mostly limited by the kinetics of the probe exchange, and complex post-acquisition data processing and a limited compatibility with live-cell imaging, because of the poor membrane permeability of the DNA strands.

#### 4.2.2. Protein and peptide ligands

Proteins constitute another useful means to provide specific reversible labelling of cellular targets. The most versatile protein binders are antibodies, but they exhibit very high affinity (nM) and thus do not really allow rapid exchange. This problem was circumvented by the use of chaotropic agent potassium thiocyanate, which disrupts the hydrogen bonds between the antigen target and Fab domain of monoclonal hemagglutinin (HA) antibody (12CA5).^53^ This low-affinity labelling with an antibody fragment in the fixed cellular environment enabled super-resolution molecular census of local populations of 3xHA tagged microtubule fibers with improved molecular quantification consistency compared to single-molecule localization microscopy (SMLM) techniques utilizing standard immunofluorescence staining for sample labelling (Fig. 6A). In another study, a specially designed buffer was reported to decrease the affinity of antibodies, which enabled further multiplexed imaging of multiple targets in cell probe exchange.^181^ Using an iterative staining–elution workflow, the authors imaged nine distinct subcellular compartments and organelles with at least seven fluorescent dyes, including the plasma membrane, actin cytoskeleton, cytoplasmic RNA, nucleoli, lysosomes, nuclear DNA, endoplasmic reticulum, mitochondria, and Golgi apparatus.

**Fig. 6:**
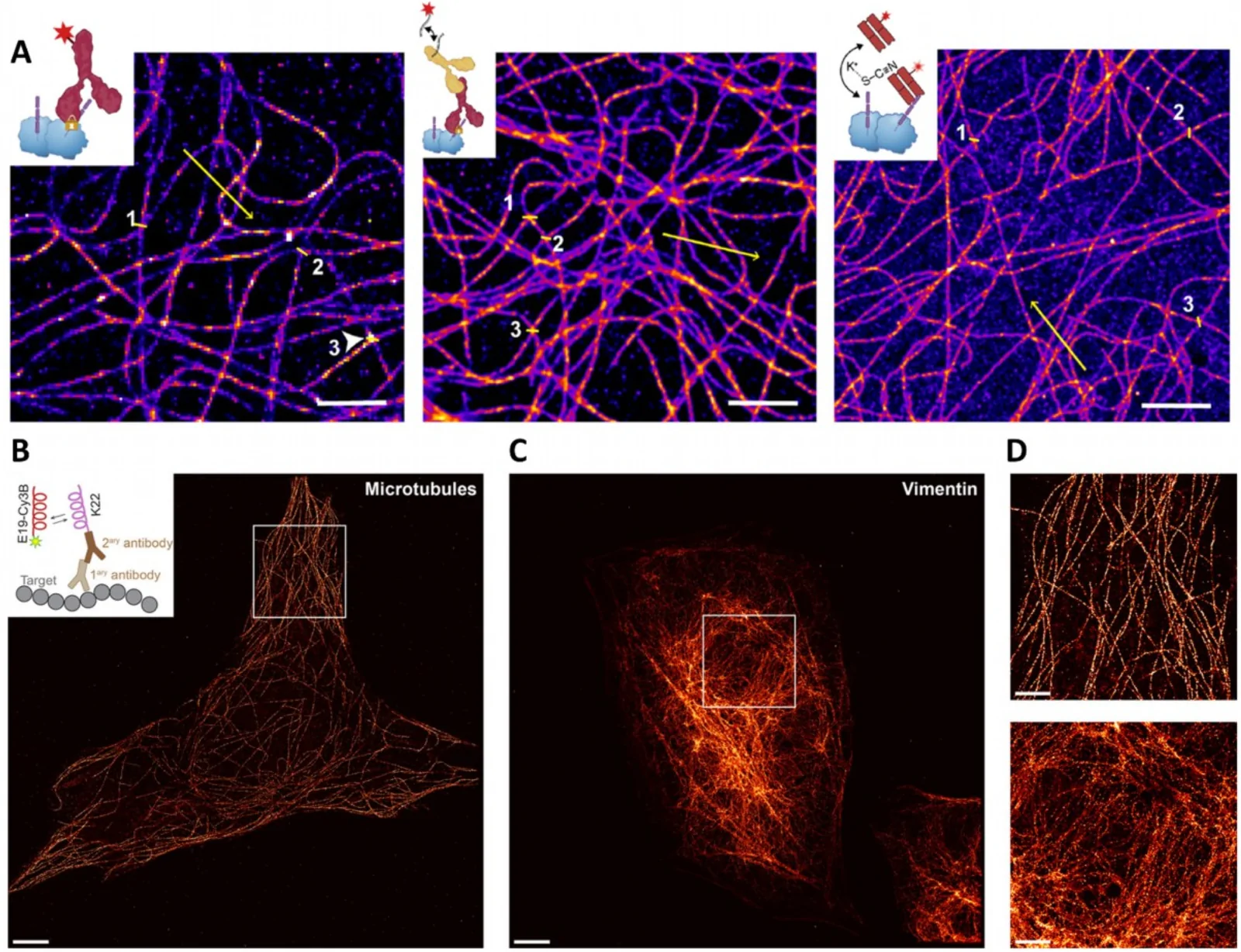
(A) Comparison of Fab-based PAINT (SR-COMET) super-resolution imaging *vs.* dSTORM based imaging on fluorescently labelled antibody and DNA-PAINT using DNA-labelled antibody, showing superior performance of the former. Reproduced from ref. 53 with permission from ACS Publications, copyright 2022. (B–D) Peptide-PAINT super-resolution imaging of microtubules and vimentin in U2OS cells. (B) Exemplary super-resolved peptide-PAINT image of microtubules labelled with primary and K22-conjugated secondary antibodies (inset: the labelling scheme). (C) Representative super-resolved peptide-PAINT data of the vimentin network. (D) Zoom-ins of highlighted areas from (B) and (C). Scale bars: 5 µm (B and C) and 2 µm (D). Reproduced from ref. 56 with permission from ACS Publications, copyright 2020.

A powerful method called IRIS was proposed based on screening well-known binders of various cellular targets, such as actin, microtubules, intermediate filaments and focal adhesions in fixed cells.^54^ The identified binders, based on protein fragments, Lifeact, a CLIP-170 fragment, a plectin-1 (PLEC) fragment and a PIPKIγ fragment, respectively, associated with and dissociated from their targets on the time scale of tens of milliseconds, which enabled PAINT type imaging of the target cellular compartments with 20 nm resolution. In another study, protein-PAINT was proposed that exploited reversible transient binding of the Src homology 2 (SH2) domain with phosphorylated tyrosine residues on receptors.^35^ This method revealed the binding sites of SH2-containing signalling molecules, such as Zap70, PI3K, Grb2, Syk, Eat2, and SHP2, to a T cell receptor. Another important example is motorPAINT, where fluorescently labelled motor proteins undergo transient binding to proteins of microtubules and undergo directional movements.^82^ This approach allows PAINT-type imaging with 52 nm resolution, revealing nanoscale organization and orientation of microtubules in neurons. However, these approaches require cell fixation.

New possibilities are offered by the use of peptide–protein and peptide–peptide interactions as a mechanism for reversible labelling. In one study, a fluorescently labelled PDZ domain was used in combination with a specific peptide genetically grafted to a target protein.^182^ It enabled 3D PAINT imaging of cytoskeletal vimentin with 53 nm resolution and the mitochondrial outer membrane transporter subunit TOMM20 with high specificity. A direct peptide analogue of DNA-PAINT was proposed by Jungmann and co-workers, called peptide-PAINT, utilizing transient coiled–coil peptide interactions, such as E- and K-coil partners.^56^ The binding kinetics of peptide-PAINT was first optimized using DNA origami nanostructures and the developed method was further applied for cellular super-resolution imaging of the microtubules and the vimentin network in fixed cells (Fig. 6B–D). Remarkably, this method was reported to outperform classical DNA-PAINT in terms of imaging speed and efficiency. Probably the shortest peptide used for PAINT-based imaging is a dipeptide diphenylalanine labelled with Cy5 dye. It was used as a PAINT probe that can reversibly co-assemble with a hydrogel formed from Fmoc-protected diphenylalanine.^58^ This approach enabled imaging of the hydrogel in 2D and 3D under its native conditions with a resolution down to ∼10 nm, showing an example of application of PAINT to biomaterials.

An important challenge is to make the PAINT approach compatible with live cell conditions, because PAINT probes, including oligonucleotides and peptides, cannot easily penetrate live cells. In this context, Regan and co-workers introduced LIVE-PAINT, where a target protein bears a short peptide tag capable of transiently binding a peptide-binding protein fused to a fluorescent protein.^175^ This approach enabled super-resolution imaging of proteins such as actin and cofilin inside live yeast cells by monitoring the stochastic association/dissociation of the protein–peptide pair. The same team further extended this approach to direct-LIVE-PAINT, which exploits a LifeAct-14 bearing GFP tag that can transiently bind to actin.^183^ This approach enabled super-resolution imaging of the dynamic nature of actin structures inside live eukaryotic cells. Thus, the problem of penetration of the PAINT probe was addressed by using endogenously produced peptide-GFP fused protein, which is a unique feature of peptide-PAINT, in contrast to classical DNA-PAINT that uses synthetic oligonucleotide–dye conjugates. However, one should note that the pool of the free probe is localized inside the cells and thus may be prone to photobleaching effects, unlike classical PAINT, where bulk buffer serves as a pool for the probe. A recent example of live cell PAINT proposed by Albertazzi and co-workers uses reversible binding of lectins to specific carbohydrate families of glyco-proteins on the cell surface, called lectin-PAINT.^184^ It enabled the identification, mapping and tracking of carbohydrates in live cells with a resolution beyond the diffraction limit as well as multiplexed 8-colour super-resolution imaging that provided glycan “fingerprints” at the single cell level.

#### 4.2.3. Other ligands

In this section we regrouped other examples that deal with other types of ligands ranging from small molecules up to relatively complex RNA aptamers, such as transient binders of biomolecular targets.

Important examples of small ligands are carbohydrates, used by Albertazzi and co-workers to introduce Glyco-PAINT.^60^ This method exploits weak glycan–lectin interactions to ensure transient binding of a fluorescently labelled carbohydrate to its receptor on the surface of live cells. Beyond super-resolution imaging, this study revealed how sugar multivalency and presentation geometry can be optimized for controlling binding kinetics and internalization.

Hu and co-workers proposed to use phalloidin–actin interactions for reversible labelling and super-resolution imaging (phalloidin-PAINT, Fig. 7A–C).^59^ As the affinity of phalloidin to actin is relatively high, two approaches were followed to increase the dissociation constant: (a) the direct effect of dye labelling, which was evaluated by testing a number of dye–phalloidin conjugates and (b) chaotropic perturbation of the binding site using KSCN salt at high concentration (300 mM). This method enabled quantitative imaging of filamentous actin (F-actin) with 52–75 nm resolution in the cell body and delicate membrane protrusions of fixed and live dendritic cells, showing superiority compared to the dSTORM imaging method.

**Fig. 7:**
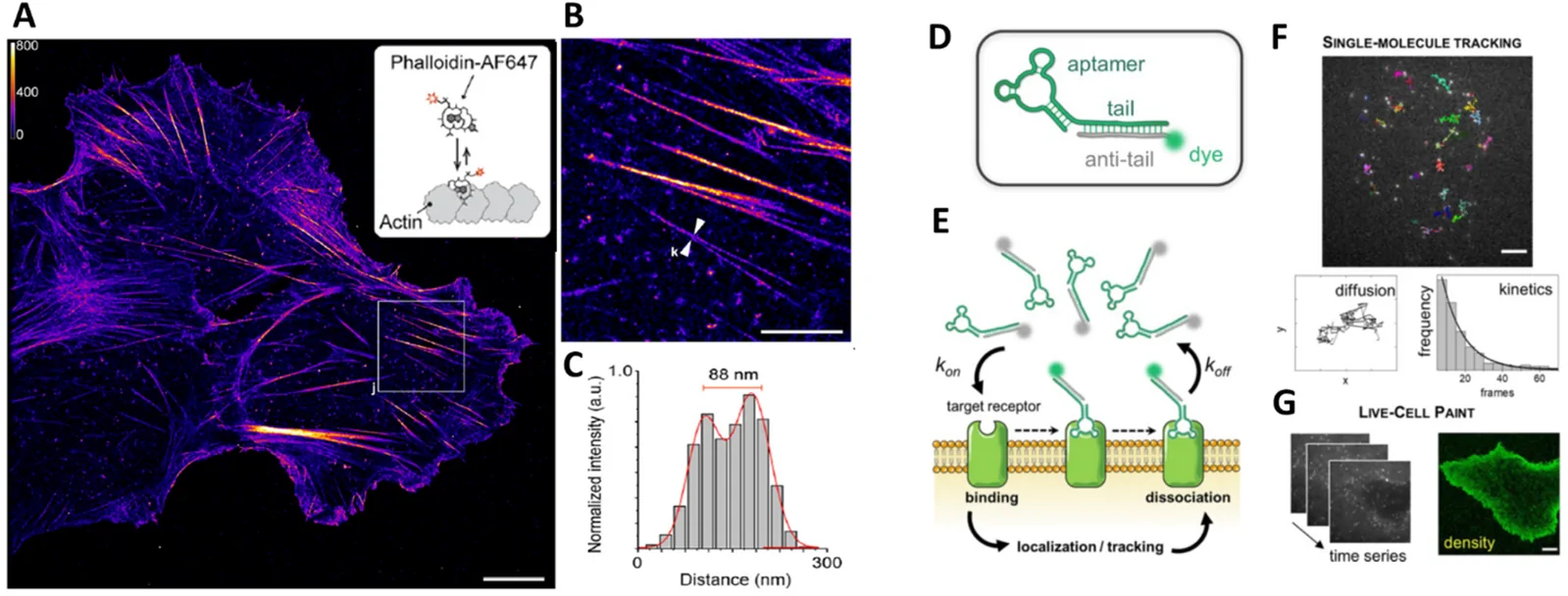
Intrinsic dissociation and single-molecule labelling with phalloidin-AF647, which enabled super-resolution imaging of F-actin. (A) Fluorescence image of the same DC2.4 cell stained with phalloidin-AF647 incubated in DPBS buffer. (B) Zoomed-in views of the boxed regions in (A). (C) Analysis of the resolution of the image in (B). Reproduced from ref. 59 with permission from Cell Press, copyright 2024. (D) The fluorescently conjugated aptamer probe. The aptamer tail (green) is annealed to its complementary anti-tail (grey) bearing a fluorophore (green sphere). (E) Schematic of live-cell imaging using aptamers. Thanks to TIR illumination, only aptamers (green fluorophores) that bind cell-surface receptors are selectively excited and detected. In contrast, freely diffusing aptamers (grey fluorophores) are not observed. Transient binding of aptamers to target receptors enables single-molecule imaging in nearly unperturbed living cells. (F) Single-molecule tracking performed using sub-nanomolar concentrations of probes (0.05–0.20 nM) to study receptor diffusion and aptamer binding kinetics. Examples of single EGFR trajectories on the surface of an A431 cell are shown (top image). Scale bar: 5 mm. *X*–*Y* coordinates of a representative trajectory are also displayed. Diffusive status of a receptor can be studied by analysis of thousands of trajectories (bottom panels). Additionally, aptamer binding kinetics can be assessed by analysing the distribution of trajectory durations, which follows a single-exponential decay (black line). (G) Membrane receptor densities obtained by live-cell PAINT using low nanomolar probe concentrations (1–20 nM). Time-lapse sequences are recorded (on the left) and then a PAINT image (on the right) is reconstructed. Scale bar: 5 mm. Reproduced from ref. 185 with permission from Wiley, copyright 2020.

Using RNA aptamers is another approach for low-affinity labelling that emerged quite recently. Albertazzi and co-workers showed that decreasing the affinity of the RNA aptamer to the epidermal growth factor receptor (EGFR) provided a higher resistance to photobleaching of the dyes used and allowed PAINT super-resolution imaging.^185^ This method enabled single-molecule tracking in order to define receptor diffusion as well as quantification of the native receptor density at the plasma membrane surface for a panel of living cancer cells (Fig. 7D–G).

An emerging opportunity is to exploit the use of dynamic covalent chemistry, which deals with reversible reactions forming unstable covalent bonds (*e.g.* imine, hydrazone, *etc.*).^186–188^ However, for the moment, it is still underexplored with only a few examples, including the use of dynamic bonds to switch ON/OFF a fluorescent dye in lipid membranes^189^ and low-affinity labelling of cell organelles using dynamic hydrazone bonds.^190^

## 5. Discussion: comparison of existing labelling methods

A summary of the existing low-affinity labelling approaches is presented in Table 1, where the examples discussed throughout this review are analysed in terms of their mechanisms of fluorogenic (turn-ON) response, biological targets, achieved spatial resolution, and compatibility with live or fixed cells. This table allows making a number of conclusions about the current state of the art in the field. Methods based on fluorophore recognition, *e.g.* probes for membranes, protein tags and RNA aptamers, systematically use environment-sensitive molecules with fluorogenic response. This constitutes an important advantage as it decreases background especially if high concentration of the probes is used. On the other hand, current environment-sensitive probes may still lack sufficient brightness and photostability. In this respect, converting high performance dyes such as rhodamine and cyanine derivatives into environment-sensitive probes is a particularly attractive approach. On the other hand, ligand-recognition techniques rely on classical always-ON dyes, probably because it is challenging to connect low-affinity ligand binding with light-up of the fluorophore in the proximity. In terms of biological targets, recent years have witnessed a dramatic expansion of low-affinity labelling strategies, which now allow the targeting of lipid membranes, DNA, proteins, peptides, carbohydrates, and many other biomolecules. However, in terms of imaging resolution DNA-PAINT is currently the most powerful method, because it enables fine tuning of ligand affinity and a very large number of binding–unbinding cycles, which is essential for RESI. Compatibility with live cell imaging remains an important limitation for low-affinity labelling methods, especially for those that require delivery of large molecules, such as DNA, peptides and proteins, inside the cells. In this respect, fluorophore-recognition methods are clearly advantageous, as they require delivery of rather small dye molecules into the cells. However, these methods generally require genetic modification in order to introduce protein or RNA tags, with the exception of membrane probes, exploiting universal dye–membrane interactions or probes for endogenous biomolecules.

**Table 1:** Summary of existing approaches for low-affinity labelling

Probe/principle/fluorophore	Turn-on mechanism	Target	Resolution (nm)	Live/fixed cells	Comments	Reference
PAINT/Nile red	ICT	Model membranes	25	—		30
SR-PAINT/Nile red	ICT	Cell membrane, ER, and mitochondria	18	Live/fixed	Spectrally resolved PAINT	39
SR-PAINT/Nile red	ICT	Cell membrane, ER, and mitochondria	30	Live/fixed	Spectrally resolved PAINT	40
SR-PAINT/NR4A/Nile red	ICT	Cell plasma membrane	<100 nm	Live	Specific targeting of PM	43
STED/NR4A/Nile red	ICT	Cell plasma membrane/endocytosis	80	Live	Reduced photo-bleaching/toxicity	29
PAINT/fluorogenic dimer/Cy3/Cy5	ACQ	Cell plasma membrane	31	Live	40 s	37
SIM/Fluorogenic dye/APBD	ICT	Mitochondria and ER	170	Live		104
STED/PAINT/xHTLs-HaloTag/SiR, JF, and TMR	RIR/SPIRO	Vimentin, mitochondria, and lysosomes	32	Live/fixed		33
FAST tag-ensconsin/fluorogen and HBR-3,5DOM	RIR	Microtubules	28	Live/fixed		111
Spinach aptamer/fluorogen/DFHBI	RIR	Intracellular RNA	Diffract. limited	Live		46
Broccoli aptamer/fluorogen/DFHBI	RIR	Intracellular RNA	Diffract. limited	Live		114
Mango aptamer/fluorogen/TO-1	RIR/ICT	Intracellular RNA	Diffract. limited	Live		116
o-Coral aptamer/fluorogenic dimer/Gemini-561	ACQ	Intracellular RNA	Diffract. limited	Live		118
biRhoBAST aptamer/fluorogenic dimer/TMR2	ACQ	Intracellular RNA	Diffract. limited	Live		121
STED/PAINT RhoBAST aptamer/fluorogenic dimer/SiR	ACQ	Intracellular RNA	80	Live/fixed		67
DNA-PAINT/Alexa655/Cy3B	None	DNA origami	80	—		31
DNA-PAINT/ATTO 655	None	DNA origami	10	—	Multiplexing	32
DNA-PAINT/Cy3B and ATTO655	None	Microtubules, mitochondria, Golgi apparatus, and peroxisomes	100	Fixed		32
DNA-PAINT/RESI/Cy3B	None	DNA origami and CD20 proteins	0.7	Fixed		22
DNA-PAINT/RESI/Cy3B and ATTO655	None	Glycans on the cell surface	0.9	Fixed		139
DNA-tkPAINT/Cy3B	FRET	Pol II S5p	3 (*x*–*y*); 8 (*z*)	Fixed + cryosectioning	3D imaging of the entire cell with higher localization precision	147
Cy3B/BHQ2						
DNA-PAINT/LD655	None	AMPAR receptors/synaptic region	30 (*x*,*y*); 75 (*z*)	Live	Limited to the cell surface	138
Repeat DNA-PAINT/ATTO 655	None	DNA origami fixed porcine cardiac tissue	12 (origami) 78 (tissue)	Fixed	10-Fold faster than original DNA-PAINT	151
FRET DNA-PAINT/ATTO488/ATTO 647N	FRET		11	Fixed	10–100-fold faster than original DNA-PAINT	153
DNA-PAINT/fluorogenic heterodimer/Cy3B/BHQ2 ATTO643/IBFQ	FRET	Microtubules, ER and mitochondria	1.7 (*x*,*y*) 4.5 (*z*)	Fixed	25.8-Fold faster than original DNA-PAINT	157
Antibody/chaotropic agent/AF647	None	Microtubules	50	Fixed		53
Dyes/antibody/special buffer/AF555 and trackers	None	Proteins/organelles	Diffract. Limited	Fixed	Multiplexing	181
IRIS/protein fragment binders/Atto488	None	Actin, microtubules, intermediate filaments/focal adhesion	20	Fixed		52
MotorPAINT/motor proteins/GFP	None	Microtubules	52	Fixed		82
PDZ domain/peptide interaction/mNeon-Green	None	Cytoskeletal vimentin/mitochondrial markers	53	Fixed		182
Peptide-PAINT/coiled–coil interactions/Cy3b	None	Microtubules/vimentin network	<20	Fixed	Faster than classical DNA-PAINT	56
Diphenylalanine/Cy5	None	Hydrogels	10	—		58
LIVE-PAINT/protein–peptide/GFP	None	Actin and cofilin		Live		175
LIVE-PAINT/LifeAct-14/GFP	None	Actin		Live	Imaging actin dynamics	81
Lectin-PAINT/lectins/Atto532/Atto643	None	Cell surface glycans	30b	Live	8-Colour multiplexing/glycan fingerprints	184
Glyco-PAINT carbohydrate–lectin/Atto565/Atto655	None	Cell surface lectins	19b	Live		60
Phalloidin-PAINT/chaotropic agent/AF647	None	F-actin	52	Fixed	Superior to dSTORM	59
RNA aptamers/Atto647N/AF488	None	Receptors on the cell surface	—	Live	Receptor diffusion/density	191

A qualitative comparison of existing low-affinity labelling methods is presented in Table 2, where the techniques are analysed in terms of major characteristics: affinity tunability, signal-to-background ratio (S/B), speed, resolution, live cell suitability, multiplexing capacity and atom economy. It highlights the current strengths and weaknesses of each method and could help biologists choose the most appropriate method and chemists focus their efforts on improving labelling strategies. Let us analyse these methods one by one.

**Table 2:** Comparative analysis of different low-affinity labelling methods^a^

Concept	Labelling method/target	Affinity tunability	S/B	Imaging speed	Imaging resolution	Live-cell suitability	Multiplexing	Atom economy	Simplicity
Fluorophore recognition	Biomembranes	++	+++	++	+	++	−	+++	+++
	Protein tags	+	++	+	+	+++	++	−	+
	RNA aptamers	+	++	+	+	+++	++	−	+
	Endogenous biomolecules	+	++	+	+	+++	++	+++	+++
Ligand recognition	DNA-PAINT	+++	+	++	+++	+	+++	−	+
	Protein/peptide ligands	+	+	++	+	+	+	−	+
	Other ligands	+	+	+	+	++	++	+++	++

a +++ is excellent, ++ is good; + is satisfactory; − is unsatisfactory.

Probes for biomembranes allow good tuning of their affinity using appropriate lipophilic chains and polar charged groups and they provide an excellent S/B ratio because of the fluorogenic nature of the dyes used. The speed is good because some probes enable rapid binding–unbinding, while the resolution remains limited because of lateral dye diffusion within the bilayer. Multiplexing is challenging, because selective and simultaneous imaging of different biomembranes remains difficult. On the other hand, the method is excellent in terms of atom economy and simplicity, because it relies on small-molecule probes and very simple and fast protocols.

Probes based on protein tags and RNA aptamers are very similar in all mentioned terms, because they share the same principle: engineering of a biomolecule tag in order to be reversibly labelled by the fluorophore. The affinity can be tuned but it requires a dedicated design of a protein or RNA tag in each case. Variation of the fluorogen structure could be an option to simplify this task. The S/B ratio of these methods is good because they generally use fluorogenic dyes, but it could still be improved. Their imaging speed remains a challenge, because it is not easy to ensure very fast dye-biomolecule exchange. Their performance in super-resolution imaging remains moderate, possibly because photodamage of the binding site by the fluorophore limits the number of binding-unbinding cycles in comparison to ligand-recognition methods (especially DNA-PAINT). On the other hand, both approaches are perfectly suitable for live cell imaging and they enable good multiplexing, because a significant number of orthogonal protein tags and RNA aptamers have already been reported to selectively bind dyes of different colours. However, their atom economy remains a weak point, because protein tags and RNA aptamers are large biomolecules, despite efforts to reduce their size. The protocols for their use remain complex, because they require transfection of the cells in order to express a protein or RNA with a corresponding tag.

The approach based on low-affinity targeting of endogenous biomolecules is still emerging, even though the first examples have been known since many years (*e.g.*, a simple example of Hoechst 33258). The major difficulty here is controlling affinity to the target, while ensuring sufficient specificity in the complex context of the cell. Generally, this method provides a good S/B ratio because of the use of fluorogenic dyes. However, speed and resolution remain challenging because of the difficulty of tuning the affinity and specificity of these probes. On the other hand, the approach is excellent for live cell imaging, because it generally uses small molecule probes. They can be combined with other probes for multiplexing, even though examples of small-molecule probes for specific low-affinity targeting of endogenous biomolecules remain limited. The approach features excellent atom economy because it is usually based on small molecules and the protocols of labelling are rather simple and fast.

DNA-PAINT is by far the most popular low-affinity labelling method to date. Its versatility stems from remarkable tunability of DNA hybridization, allowing design of oligonucleotides with finely tuned affinity and high specificity. Even though it is generally rather slow, its speed was significantly improved through design of new hybridization schemes. It also features outstanding resolution due to the use of the RESI approach, owing to the almost unlimited number of binding-unbinding events, which are rather decoupled from fluorophore photochemistry. Owing to great variability of DNA sequences, it provides outstanding capacity for multiplexing both using labels of different colours and multiple stain–wash cycles. On the other hand, it has a number of major weaknesses; in particular, its S/B ratio remains limited, because it is difficult to couple hybridization with dye light up, although FRET-based and quencher-based fluorogenic imagers have recently been developed to address this issue. The key limitation of DNA-PAINT is its incompatibility with intracellular live-cell imaging, because it uses oligonucleotides in combination with antibodies or other targeting ligands, which are cell impermeable. Therefore, current examples of live cell imaging with DNA-PAINT are limited to the cell surface. The atom economy of this method is a weak point as it uses relatively large targeting ligands in combination with oligonucleotides. Therefore, high-resolution with DNA-PAINT was achieved only when the oligonucleotide–dye conjugate was positioned rather precisely at the site of interest. Finally, the method is not straightforward as it generally requires synthesis of biomolecule-oligonucleotide conjugates and specially designed dye-labelled oligonucleotides.

The use of peptide/protein ligands in low-affinity labelling is an emerging approach and its performance therefore remains moderate for most of the above characteristics compared with DNA-PAINT. Nevertheless, it has some unique features that could provide advantages. For example, some peptides could be able to penetrate the cells. Moreover, peptide–protein interaction could be exploited to label live cells expressing the protein of interest with a protein or peptide tag. Much more efforts will be needed in this direction. Low-affinity labelling using other ligands offers multiple new opportunities compared to DNA-PAINT and peptide-PAINT. The use of small molecule ligands featuring excellent atom economy enables compatibility with live cell imaging and more precise positioning of the dye at the labelling site, which is important for super-resolution imaging. It has high potential in terms of multiplexing, because of a large variety of existing small molecule ligands for multiple biomolecular targets. This method can be directly used to label endogenous biomolecules (*e.g.* endogenous receptors) without genetic modification, making the protocols of staining rather easy. Nevertheless, this approach remains underdeveloped and future efforts should be directed to tune affinity and ensure fluorogenic response as well as to achieve high speed and resolution of corresponding imaging methods.

## 6. Conclusions and perspectives

Classical fluorescence-labeling approaches are based on the covalent conjugation of a dye to the target biomolecule or on high-affinity probe–target interactions. However, recent studies have shown that low-affinity labeling can represent an attractive alternative with several major advantages. The primary advantage is the continuous exchange of labels at the target site, which enables virtually unlimited observation times with minimal photobleaching. This feature is particularly important for long-term imaging and single-molecule tracking. In addition, low affinity generally results in a low occupancy of binding sites, which may reduce phototoxicity. This aspect is especially relevant for advanced high-resolution techniques, such as STED, which requires high excitation power densities. Finally, reversible low-affinity labeling is central to PAINT super-resolution imaging, where binding events are directly associated with fluorophore immobilization and/or fluorescence light up. Rapid dye exchange also enables multiplexed imaging of a large number of targets using successive staining and washing cycles. DNA-PAINT has demonstrated that a large number of targets can be sequentially addressed and the limitation originates only from the number of orthogonal DNA sequences that can be generated from relatively short imaging and docking strands. Reversible labeling is also important in biosensing applications, for example in aptamer-based sensors, where binding and unbinding events are controlled by analyte recognition.

Despite the great diversity of low-affinity labeling strategies, several important challenges remain. The first is specificity, which can be compromised by the inherently low affinity of the interaction. In DNA-PAINT, specificity is relatively straightforward to achieve because it is ensured by sequence complementarity, while affinity can be tuned through the length of the hybridizing strands. In contrast, achieving both specificity and appropriate affinity is considerably more challenging for other probe–target interactions and often requires dedicated molecular design, such as the engineering of membrane-binding probes, proteins, or aptamers. The second challenge concerns exchange kinetics. Ideally, probe exchange should be sufficiently rapid to ensure continuous replenishment of fluorophores while remaining slow enough to allow reliable detection of individual binding events. Several solutions have been proposed in the DNA-PAINT field, including repeat-PAINT and FRET-PAINT, which accelerate imaging while reducing background fluorescence. Third, the majority of exchangeable probes still rely on always-ON fluorescent dyes, whereas fluorogenic probes would be highly advantageous. Consequently, recent years have seen the emergence of fluorogenic low-affinity probes based on dye dimers and specially engineered fluorogenic dyes. Efficient ON/OFF fluorescence switching is particularly useful for minimizing background signals and enabling the use of higher probe concentrations. This strategy provides an additional route toward faster super-resolution imaging, as demonstrated for FRET-PAINT and fluorogenic dimer probes targeting lipid membranes. The fourth challenge is cell permeability to the probes, since live-cell imaging requires that probes efficiently access intracellular targets. This issue is particularly important for DNA hybridization-based methods because DNA–dye conjugates are generally cell-impermeable, restricting most applications to fixed cells or targets located on the surface of living cells. Therefore, DNA-PAINT is still predominantly applied to fixed-cell imaging. In contrast, emerging live-cell imaging strategies based on reversible labeling include genetically encoded protein tags and fluorogenic probes targeting endogenous biomolecules or cellular membranes. Finally, phototoxicity and photodamage remain important concerns. Long-term imaging requires fluorophores with minimal phototoxicity to fully exploit the benefits of continuous label exchange. Furthermore, repeated excitation of exchangeable fluorophores may induce photodamage at the binding site itself, potentially affecting the integrity of the targeted biomolecule.

In addition to improving resolution and increasing observation time, low-affinity labelling modifies the type of information that super-resolution microscopy can provide. As each binding event involves an independent single-molecule measurement using a fresh, non-bleached probe, additional parameters can be added to each localization, such as the emission spectrum, residence time, diffusion coefficient or orientation of the probe. When combined with environment-sensitive fluorogenic dyes, single-molecule localization microscopy becomes a multiparametric approach that not only maps where a target is located, but also sheds light on its local physicochemical environment (*e.g.* polarity, lipid order and viscosity), mobility and molecular organization. This has already been illustrated in Section 3.1 using SR-PAINT, diffusivity mapping and orientation imaging of cell membranes. The binding kinetics themselves also carry functional information: residence times and association rates measured at individual sites provide information about the local affinity, accessibility, or conformational state of the target. They can also serve as kinetic barcodes for multiplexing. A key limitation of this strategy is the photon budget, as each additional dimension competes with the localization itself because of the limited number of photons collected per binding event. Brighter probes with well-controlled affinity and residence times will therefore be essential. An additional dimension can be provided by molecular probes with affinity, exchange kinetics or emission properties controlled by defined biological parameters such as membrane potential, pH, ion concentrations, mechanical tension, or local biophysical properties, like polarity and viscosity. When coupled with spectral and polarization-resolved detection, fast cameras and machine learning analysis, these probes could transform super-resolution microscopy from ‘seeing structures' to ‘sensing functions', enabling the quantitative nanoscale mapping of the physicochemical properties and dynamics underlying biological function in living cells.

At present, most low-affinity labeling strategies are applied to PAINT-based super-resolution imaging and single-molecule tracking. However, increasing efforts are being devoted to extending these concepts to other areas of biological research, including the study of biomolecular interactions and dynamics. Moreover, low-affinity labeling could potentially be adapted to more conventional imaging modalities, such as confocal, two-photon, and wide-field fluorescence microscopy, as well as to other super-resolution techniques including STED and structured illumination microscopy. We anticipate that this field will continue to expand rapidly, driven on the one hand by innovative concepts in fluorogenic dye design and reversible labeling strategies and, on the other hand, by the growing range of applications in advanced fluorescence microscopy.

## Author contributions

ASK proposed the concept of the review and wrote several of its chapters. RP and PD wrote the other chapters. All authors reviewed and edited the whole manuscript.

## Conflicts of interest

Some fluorescent probes (NR12S, NR12A and NR4A) mentioned in this work were commercialized by Cytoskeleton Inc. under a license from SATT Conectus/University of Strasbourg. The authors declare no other conflicts of interest.

## Data Availability

No primary research results, software or code have been included and no new data were generated or analysed as part of this review.
